# Supplementary material for: Understanding ethnic inequalities in hearing health in the UK: a cross-sectional study of the link between language proficiency and performance on the Digit Triplet Test
Source: BMJ Open. 2020 Dec 8;10(12):e042571. doi: 10.1136/bmjopen-2020-042571 (PMC7725084; doi:10.1136/bmjopen-2020-042571)
Supplement: Supplementary data [file bmjopen-2020-042571supp001.pdf]

Test centre	Average SRT Mean	Standard Deviation
Birmingham	-7.1	1.8
Bristol	-7.1	1.6
Croydon	-7.0	1.8
Hounslow	-7.4	1.9
Liverpool	-7.5	1.7
Middlesbrough	-7.5	1.9
Nottingham	-6.8	1.9
Sheffield	-7.7	1.6
Swansea	-6.0	1.8
Wrexham	-6.0	1.9
