# Supplementary figures and images for: Understanding ethnic inequalities in hearing health in the UK: a cross-sectional study of the link between language proficiency and performance on the Digit Triplet Test
Source: BMJ Open. 2020 Dec 8;10(12):e042571. doi: 10.1136/bmjopen-2020-042571 (PMC7725084; doi:10.1136/bmjopen-2020-042571)

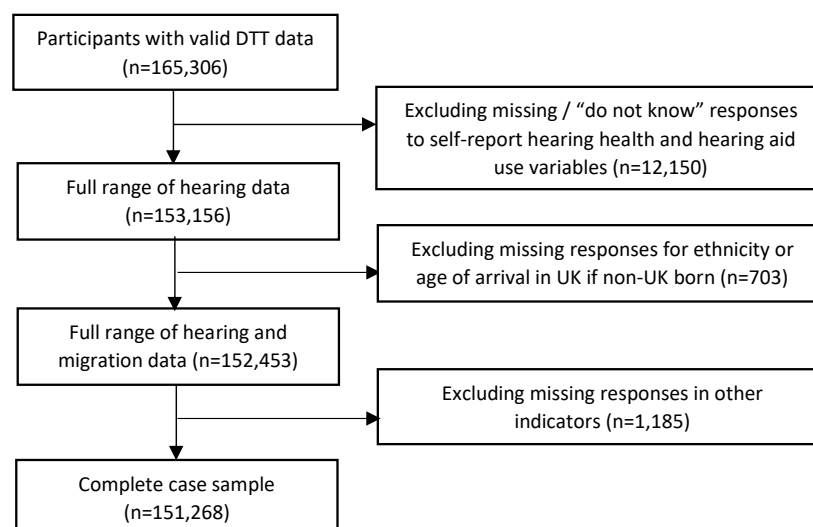

Supplement: Supplementary data [file bmjopen-2020-042571supp002.pdf]

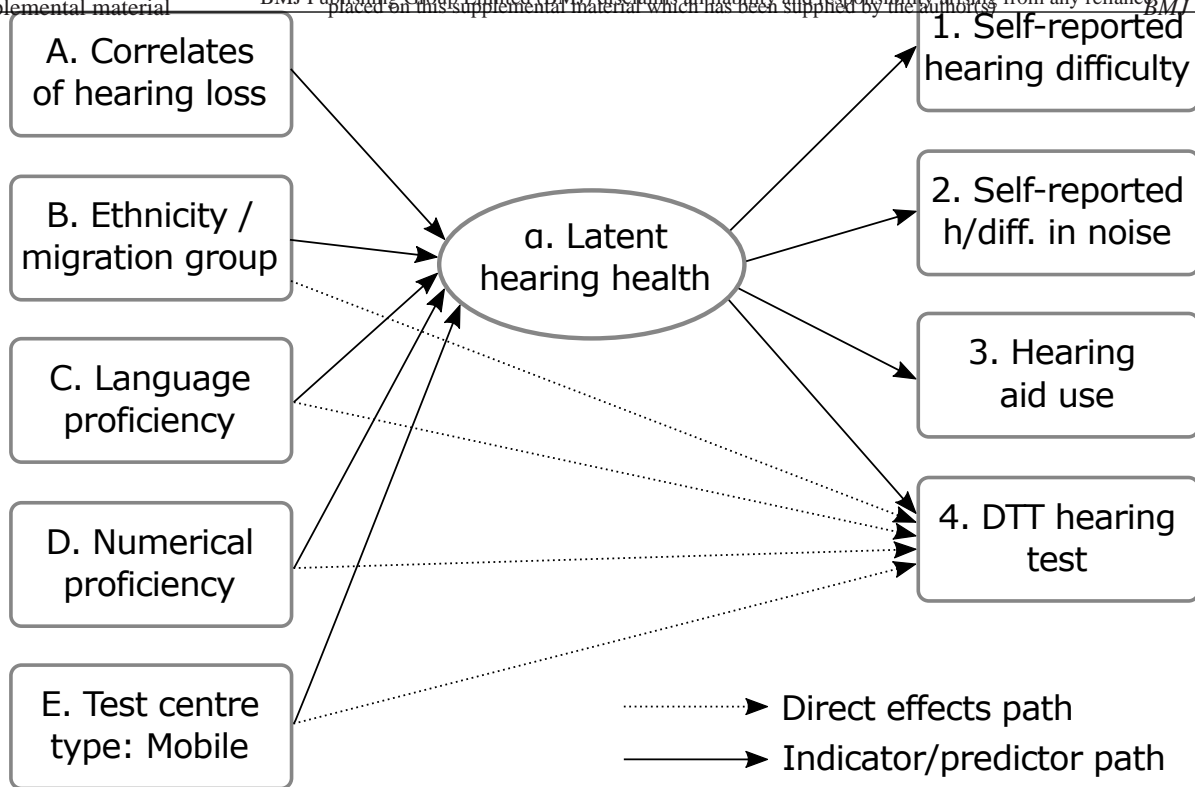

Supplement: Supplementary data [file bmjopen-2020-042571supp007.pdf]
