# Supplementary material for: Understanding ethnic inequalities in hearing health in the UK: a cross-sectional study of the link between language proficiency and performance on the Digit Triplet Test
Source: BMJ Open. 2020 Dec 8;10(12):e042571. doi: 10.1136/bmjopen-2020-042571 (PMC7725084; doi:10.1136/bmjopen-2020-042571)
Supplement: Supplementary data [file bmjopen-2020-042571supp004.pdf]

	m1	m2	m3	m4	m5
**Covariate**	**Est**	**Est**	**Est**	**Est**	**Est**
Age	1.03 (1.03 to 1.03)	1.03 (1.03 to 1.03)	1.04 (1.04 to 1.04)	1.04 (1.04 to 1.04)	1.03 (1.03 to 1.04)
Sex: Male	1.71 (1.66 to 1.75)	1.69 (1.64 to 1.74)	1.36 (1.32 to 1.41)	1.36 (1.32 to 1.41)	1.37 (1.33 to 1.41)
BME (NEEM)	0.88 (0.82 to 0.95)	0.86 (0.80 to 0.92)	0.86 (0.80 to 0.92)	0.86 (0.80 to 0.93)	0.84 (0.78 to 0.91)
BME, later-migrating	0.90 (0.85 to 0.95)	0.86 (0.81 to 0.91)	0.91 (0.86 to 0.97)	0.94 (0.89 to 1.00)	0.94 (0.88 to 1.00)
Mobile test centre	1.00 (0.90 to 1.12)	1.00 (0.90 to 1.11)	0.98 (0.88 to 1.09)	0.98 (0.88 to 1.09)	1.00 (0.90 to 1.12)
Education: High School		0.88 (0.84 to 0.93)	0.99 (0.94 to 1.04)	0.97 (0.92 to 1.02)	1.00 (0.95 to 1.05)
Education: Degree		0.99 (0.95 to 1.03)	1.13 (1.08 to 1.17)	1.10 (1.05 to 1.15)	1.14 (1.09 to 1.20)
Townsend dep. Score		1.02 (1.01 to 1.02)	1.00 (1.00 to 1.01)	1.01 (1.00 to 1.01)	1.00 (1.00 to 1.01)
Exposure to loud noise			1.84 (1.78 to 1.91)	1.85 (1.78 to 1.91)	1.85 (1.78 to 1.91)
Exposure to loud music			1.82 (1.74 to 1.89)	1.82 (1.75 to 1.89)	1.81 (1.74 to 1.89)
Use of ototoxic meds.			1.05 (1.01 to 1.10)	1.05 (1.01 to 1.10)	1.06 (1.01 to 1.11)
Stroke			1.07 (0.94 to 1.21)	1.07 (0.94 to 1.22)	1.00 (0.87 to 1.14)
Diabetes			1.03 (0.96 to 1.10)	1.03 (0.97 to 1.10)	1.02 (0.95 to 1.09)
Cardiovascular illness			1.12 (1.06 to 1.19)	1.12 (1.06 to 1.19)	1.13 (1.07 to 1.19)
Hypertension			0.93 (0.90 to 0.96)	0.93 (0.90 to 0.96)	0.93 (0.90 to 0.95)
Smoker (at some stage)			1.11 (1.07 to 1.14)	1.11 (1.07 to 1.14)	1.11 (1.07 to 1.14)
Alcohol: Former			1.10 (1.01 to 1.20)	1.10 (1.01 to 1.20)	1.08 (0.99 to 1.18)
Alcohol: Meets guidelines			1.00 (0.95 to 1.05)	0.99 (0.94 to 1.04)	1.01 (0.96 to 1.06)
Alcohol: Exceeds g/l			1.02 (0.96 to 1.07)	1.01 (0.96 to 1.06)	1.02 (0.97 to 1.08)
Meningitis as child			1.62 (1.12 to 2.35)	1.62 (1.11 to 2.35)	1.62 (1.13 to 2.32)
MMR as child			1.02 (0.90 to 1.16)	1.02 (0.89 to 1.16)	0.98 (0.86 to 1.12)
Language score: 1 correct				1.01 (0.95 to 1.08)	1.00 (0.94 to 1.06)
Language score: 2 correct				1.05 (1.00 to 1.11)	1.04 (0.98 to 1.09)
Language score: Not taken				0.91 (0.81 to 1.03)	0.89 (0.79 to 1.00)
Numeric score: >50% correct				1.04 (1.00 to 1.09)	1.04 (0.99 to 1.09)
Numeric score: Not taken				1.05 (0.94 to 1.17)	1.08 (0.97 to 1.20)
