# Supplementary material for: Understanding ethnic inequalities in hearing health in the UK: a cross-sectional study of the link between language proficiency and performance on the Digit Triplet Test
Source: BMJ Open. 2020 Dec 8;10(12):e042571. doi: 10.1136/bmjopen-2020-042571 (PMC7725084; doi:10.1136/bmjopen-2020-042571)
Supplement: Supplementary data [file bmjopen-2020-042571supp005.pdf]

Covariate	m1 Est	m2 Est	m3 Est	m4 Est	m5 Est
Age	1.13 (1.12 to 1.14)	1.13 (1.12 to 1.13)	1.13 (1.13 to 1.14)	1.13 (1.13 to 1.14)	1.15 (1.15 to 1.16)
Sex: Male	1.93 (1.82 to 2.06)	1.93 (1.81 to 2.06)	1.36 (1.27 to 1.46)	1.36 (1.26 to 1.46)	1.33 (1.25 to 1.42)
BME (NEEM)	0.74 (0.58 to 0.94)	0.72 (0.56 to 0.91)	0.68 (0.54 to 0.87)	0.67 (0.53 to 0.86)	0.83 (0.68 to 1.03)
BME, later-migrating	0.58 (0.50 to 0.68)	0.55 (0.47 to 0.65)	0.55 (0.47 to 0.65)	0.52 (0.44 to 0.62)	0.57 (0.49 to 0.67)
Mobile test centre	1.13 (0.90 to 1.41)	1.12 (0.90 to 1.40)	1.05 (0.84 to 1.32)	1.05 (0.83 to 1.32)	0.94 (0.76 to 1.17)
Education: High School		0.63 (0.57 to 0.69)	0.80 (0.72 to 0.88)	0.83 (0.75 to 0.91)	0.80 (0.73 to 0.87)
Education: Degree		0.71 (0.66 to 0.77)	0.90 (0.83 to 0.98)	0.94 (0.87 to 1.03)	0.86 (0.80 to 0.93)
Townsend dep. Score		1.03 (1.02 to 1.04)	1.01 (0.99 to 1.02)	1.01 (0.99 to 1.02)	1.02 (1.01 to 1.03)
Exposure to loud noise			2.89 (2.69 to 3.10)	2.86 (2.67 to 3.07)	2.77 (2.61 to 2.95)
Exposure to loud music			2.15 (1.95 to 2.37)	2.14 (1.94 to 2.36)	2.13 (1.95 to 2.32)
Use of ototoxic meds.			1.16 (1.06 to 1.27)	1.16 (1.06 to 1.27)	1.13 (1.05 to 1.22)
Stroke			1.15 (0.93 to 1.42)	1.14 (0.92 to 1.42)	1.33 (1.11 to 1.60)
Diabetes			1.21 (1.07 to 1.36)	1.20 (1.07 to 1.36)	1.24 (1.12 to 1.37)
Cardiovascular illness			1.24 (1.12 to 1.37)	1.24 (1.12 to 1.37)	1.20 (1.10 to 1.31)
Hypertension			0.93 (0.87 to 1.00)	0.93 (0.87 to 1.00)	0.95 (0.89 to 1.01)
Smoker (at some stage)			1.13 (1.06 to 1.21)	1.14 (1.06 to 1.21)	1.13 (1.07 to 1.20)
Alcohol: Former			1.04 (0.87 to 1.25)	1.05 (0.87 to 1.26)	1.14 (0.97 to 1.33)
Alcohol: Meets guidelines			1.01 (0.90 to 1.12)	1.01 (0.91 to 1.13)	0.95 (0.87 to 1.05)
Alcohol: Exceeds g/l			0.81 (0.72 to 0.91)	0.82 (0.73 to 0.92)	0.79 (0.72 to 0.88)
Meningitis as child			2.93 (1.59 to 5.41)	2.95 (1.60 to 5.43)	2.88 (1.64 to 5.08)
MMR as child			1.12 (0.84 to 1.49)	1.13 (0.84 to 1.51)	1.29 (1.01 to 1.65)
Language score: 1 correct				0.92 (0.81 to 1.05)	0.98 (0.87 to 1.10)
Language score: 2 correct				0.85 (0.77 to 0.95)	0.91 (0.83 to 1.00)
Language score: Not taken				0.86 (0.67 to 1.11)	0.94 (0.76 to 1.17)
Numeric score: >50% correct				1.02 (0.93 to 1.13)	1.05 (0.96 to 1.15)
Numeric score: Not taken				1.22 (0.97 to 1.53)	1.10 (0.90 to 1.35)
