# Supplementary material for: Understanding ethnic inequalities in hearing health in the UK: a cross-sectional study of the link between language proficiency and performance on the Digit Triplet Test
Source: BMJ Open. 2020 Dec 8;10(12):e042571. doi: 10.1136/bmjopen-2020-042571 (PMC7725084; doi:10.1136/bmjopen-2020-042571)
Supplement: Supplementary data [file bmjopen-2020-042571supp006.pdf]

	m1	m2	m3	m4	m5
**DTT pathway**	**Est**	**Est**	**Est**	**Est**	**Est**
BME, migrated age <12					1.04 (0.95 to 1.15)
BME, migrated age >= 12					3.85 (3.64 to 4.07)
Language score: 1 correct					0.69 (0.65 to 0.74)
Language score: 2 correct					0.61 (0.58 to 0.65)
Language score: Not taken					1.12 (1.00 to 1.26)
Numeric score: >50% correct					0.71 (0.67 to 0.75)
Numeric score: Not taken					1.34 (1.20 to 1.49)
Mobile test centre					5.52 (4.99 to 6.10)
