# Supplementary material for: Understanding ethnic inequalities in hearing health in the UK: a cross-sectional study of the link between language proficiency and performance on the Digit Triplet Test
Source: BMJ Open. 2020 Dec 8;10(12):e042571. doi: 10.1136/bmjopen-2020-042571 (PMC7725084; doi:10.1136/bmjopen-2020-042571)
Supplement: Author's manuscript [file bmjopen-2020-042571.draft_revisions.pdf]

BMJ Open is committed to open peer review. As part of this commitment we make the peer review history of every article we publish publicly available.

When an article is published we post the peer reviewers' comments and the authors' responses online. We also post the versions of the paper that were used during peer review. These are the versions that the peer review comments apply to.

The versions of the paper that follow are the versions that were submitted during the peer review process. They are not the versions of record or the final published versions. They should not be cited or distributed as the published version of this manuscript.

BMJ Open is an open access journal and the full, final, typeset and author-corrected version of record of the manuscript is available on our site with no access controls, subscription charges or pay-per-view fees (http://bmjopen.bmj.com).

If you have any questions on BMJ Open's open peer review process please email info.bmjopen@bmj.com

BMJ Open

Understanding hearing health inequalities in ethnic minority groups in the UK

Journal:	*BMJ Open*
Manuscript ID	bmjopen-2020-042571
Article Type:	Original research
Date Submitted by the Author:	22-Jul-2020
Complete List of Authors:	Taylor, Harry; The University of Manchester School of Social Sciences, Social Statistics; The University of Manchester Cathie Marsh Institute for Social Research<br>Shryane, Nick; The University of Manchester School of Social Sciences, Social Statistics; The University of Manchester Cathie Marsh Institute for Social Research<br>Kapadia, Dharmi; The University of Manchester School of Social Sciences, Sociology; The University of Manchester Cathie Marsh Institute for Social Research<br>Dawes, Piers; The University of Manchester School of Health Sciences, Manchester Centre for Audiology and Deafness; Manchester University NHS Foundation Trust, Manchester Academic Health Science Centre<br>Norman, Paul; University of Leeds, School of Geography
Keywords:	EPIDEMIOLOGY, Audiology < OTOLARYNGOLOGY, STATISTICS & RESEARCH METHODS

SCHOLARONE™
Manuscripts

I, the Submitting Author has the right to grant and does grant on behalf of all authors of the Work (as defined in the below author licence), an exclusive licence and/or a non-exclusive licence for contributions from authors who are: i) UK Crown employees; ii) where BMJ has agreed a CC-BY licence shall apply, and/or iii) in accordance with the terms applicable for US Federal Government officers or employees acting as part of their official duties; on a worldwide, perpetual, irrevocable, royalty-free basis to BMJ Publishing Group Ltd ("BMJ") its licensees and where the relevant Journal is co-owned by BMJ to the co-owners of the Journal, to publish the Work in this journal and any other BMJ products and to exploit all rights, as set out in our licence.

The Submitting Author accepts and understands that any supply made under these terms is made by BMJ to the Submitting Author unless you are acting as an employee on behalf of your employer or a postgraduate student of an affiliated institution which is paying any applicable article publishing charge ("APC") for Open Access articles. Where the Submitting Author wishes to make the Work available on an Open Access basis (and intends to pay the relevant APC), the terms of reuse of such Open Access shall be governed by a Creative Commons licence – details of these licences and which Creative Commons licence will apply to this Work are set out in our licence referred to above.

Other than as permitted in any relevant BMJ Author's Self Archiving Policies, I confirm this Work has not been accepted for publication elsewhere, is not being considered for publication elsewhere and does not duplicate material already published. I confirm all authors consent to publication of this Work and authorise the granting of this licence.

For peer review only

Understanding hearing health inequalities in ethnic minority groups in the UK

Corresponding author:

Harry Taylor, Room G45, Humanities Bridgeford Street, Oxford Road, Manchester, M13 9PL. harry.taylor-2@postgrad.manchester.ac.uk, +44 7751 220823.

Co-authors:

1. **Nick Shryane**, The Department of Social Statistics, The University of Manchester, Manchester, UK.
   also The Cathie Marsh Institute for Social Research (CMIST), The University of Manchester, Manchester, UK.
2. **Dharmi Kapadia**, The Cathie Marsh Institute for Social Research (CMIST), The University of Manchester, Manchester, UK.
3. **Piers Dawes**, Manchester Centre for Audiology and Deafness, School of Health Sciences, University of Manchester, Manchester, UK;
   also Manchester University NHS Foundation Trust (MFT), Manchester Academic Health Science Centre, Manchester, UK;
   also Department of Linguistics, Macquarie University, Sydney, Australia;
4. **Paul Norman**, School of Geography, University of Leeds, Leeds, UK

For peer review only

ABSTRACT

Introduction

Research using UK Biobank data has shown ethnic inequalities in hearing health; however, the hearing test used may exhibit a disadvantage for non-native language speakers.

Objectives

To validate the results of the UK Biobank hearing test (Digit Triplet Test, DTT) against self-reported measures of hearing in the dataset and create classifications of hearing health. To observe if language proficiency and migration age have the same effect upon hearing health classification as upon the DTT in isolation. Our hypothesis is that language proficiency acts differently upon the DTT, demonstrating that the DTT is biased for non-native speakers of English.

Design

Latent classes representing profiles of hearing health were identified from the available hearing measures. Factors associated with class membership were tested using multinomial logistic regression models. Ethnicity was defined as i) White, native English-speaking, ii) ethnic minority, arrived in UK aged <12 or iii) ethnic minority, arrived aged =>12.

Participants

UK Biobank participants with valid hearing test results and associated covariates (N=151,271)

Outcome measures

DTT score, self-reported hearing difficulty, self-reported hearing difficulty in noise and hearing aid use

Results

Three classes of hearing health were found, 'Normal', 'Generally Poor' and 'Only Subjectively Poor'. In a model adjusting for known confounders of hearing loss, a poor or insufficient hearing test result was less likely for those with better language (OR 0.69, 0.65-0.74 95% CI) or numerical ability (OR 0.71, 0.67-0.75 95% CI) but more likely for those having migrated aged >= 12 (OR 3.85, 3.64-4.07 95% CI).

Conclusions

The DTT showed evidence of bias, having greater dependence upon language ability and migration age than other hearing indicators. Designers of future surveys and hearing screening applications may wish to consider the limitations of speech-in-noise tests in evaluating hearing acuity for populations that include non-native speakers.

For peer review only

ARTICLE SUMMARY

Strengths and limitations of this study

- To our knowledge, the first study comparing subjective and objective hearing outcomes for ethnic minorities using UK Biobank data
- Potential language bias in the UK Biobank's hearing test was investigated for the first time
- All available hearing outcomes from the UK Biobank were combined into 3 latent classes; the behaviour of these classes was substantively consistent with the literature
- This study uses the available language proficiency data in the UK Biobank, which is limited.

For peer review only

INTRODUCTION

Hearing impairment is one of the most common chronic age-related health conditions [1]; it is estimated that 1 in 5 people in the UK will have hearing loss, characterised by 25dB or more hearing loss in the better ear, by 2035 [2]. Ethnic inequalities in health outcomes are well-documented and have been a key focus for UK government over the last 25 years [3]. Yet research into ethnic inequalities in hearing health in the UK has been limited due to insufficient ethnic minority sample sizes.

This research study is concerned with the ethnic inequality observed by Dawes and colleagues while investigating the correlates of hearing loss using UK Biobank data [4]. The study found that people who reported a Non-White ethnic background were 3.27 (3.07-3.48, 95% CI) times more likely to have hearing loss than those who identified as White British. This result was consistent with reports of higher levels of other chronic health conditions among minority groups in the UK [5], but contradicted observations from the USA where hearing loss was 70% lower in Black groups compared to White groups [6].

Hearing acuity in the UK Biobank is measured using the Digit Triplet Test (DTT), a test of speech recognition that involves perception of three spoken digits. Other UK health surveys collect hearing data using methods based on pure-tone audiometry (PTA) [7,8], a non-language-based evaluation of hearing acuity employing detection of pure tones. The DTT generally shows good correlation with PTA measures [9]; however, the word-based approach of speech-in-noise tests measures language comprehension as well as hearing acuity. This could present a scenario whereby a poor test result for a non-native speaker is due not to poor hearing, but to a lower level of language proficiency compared with native speakers [10]. This language penalty has been shown to increase the more linguistically-complex the speech-in-noise test [11].

Given the conflation of language ability and hearing acuity in the DTT, the present study divides the ethnic minority cohort into those likely to have native-level English proficiency, and those who migrated to the UK as an adolescent or older. It is hypothesised that, correcting for respondents' self-reported hearing health and related factors, DTT scores for the older-migrating cohort will be worse due to their likely lower language proficiency.

The present research therefore aims to validate the results of the DTT against three self-reported measures of hearing provided in the UK Biobank. These measures will be used to create classifications of hearing health. We will see if language proficiency and migration age have the same effect upon the hearing health classifications as for the DTT in isolation.

Our hypothesis is that the DTT is biased for non-native speakers of English. If the DTT is not biased, it would be expected that language test scores would have the same effect across every measure of hearing in the dataset. A different outcome would indicate a dependency on language

For peer review only - http://bmjopen.bmj.com/site/about/guidelines.xhtml

ability. If, after controlling for language ability, the DTT still shows poorer outcomes for the ethnic minority groups, it can be concluded that inequalities are present regardless of any language bias.

METHOD

Sample and design

The study population was participants in the UK Biobank, a biomedical study of over 500,000 adults aged 40-69 recruited in the United Kingdom from 2006-2010 [12]. Participants were invited to the study based on their proximity to one of 25 test centres and being registered with the National Health Service [12]. The study design was cross-sectional, using only the first wave of UK Biobank responses. The present research considers the 151,271 adults who produced valid DTT results and had a complete set of variables.

Measures

Hearing health

Four measures of hearing health were used to form the hearing health classifications: the DTT, two self-reported measures of hearing difficulty, and self-reported hearing aid use. All data, including the objective measure, were collected in a self-administered manner via the touchscreen questionnaire undertaken in Biobank test centres.

As a speech-in-noise test, the DTT was devised to detect impaired speech intelligibility in noisy conditions [13], which is one of the most frequently-reported hearing difficulties associated with hearing loss [14]. The DTT was originally validated using participants whose first language was English [15]. The test produces a Speech Recognition Threshold (SRT) measured using signal to noise ratio in decibels (dB) [13], with lower scores representing better performance. The lowest score in the better ear was used in the present study, with scores above -5.5dB characterised as 'insufficient/poor' as per Dawes et al. [4]. Respondents who stated they were completely deaf or used a cochlear implant were excluded due to having no DTT data.

For the self-reported measures, respondents were asked: "Do you have any difficulty with your hearing?" (*Yes / No / I am completely deaf / Do not know / Prefer not to answer),* and "Do you find it difficult to follow a conversation if there is background noise (such as TV, radio, children playing)?" (*Yes / No / Do not know / Prefer not to answer)*. The completely deaf category was amalgamated with the "Yes" category. Respondents were asked if they currently use a hearing aid "most of the time" *(Yes / No / Prefer not to answer)*. Participants responding "Do not know" or "Prefer not to answer" were excluded from the dataset.

For peer review only - http://bmjopen.bmj.com/site/about/guidelines.xhtml

Correlates of hearing loss

Hearing health is affected by a number of demographic, socioeconomic and lifestyle factors, as well as co-morbidity from associated diseases [6]. These factors were controlled for in the final analysis.

Hearing loss is more likely for males, and with increasing age [16,17]. Socioeconomic status has been shown to affect hearing, including education [18] and income [19]. Education was coded as either no formal qualification, secondary-school level qualifications or any further education. The Townsend index, an area-level deprivation measure, was used instead of household income, which had a high number of missing values.

Exposure to noise is a well-established factor in hearing loss [6]. Measures were included for whether the participant had ever worked in an environment where they had to shout to be heard, or if they had previously been exposed to loud music.

Lifestyle factors known to affect hearing were included. Smoking [6] was categorised as never smoker or former/current smoker. Alcohol consumption [20] was calculated based on estimated weekly intake per type of drink, and an ethanol content estimate for each type. Where an estimate was unavailable, a predicted value was assigned according to alcohol intake frequency. The resulting intake was categorised against the 112g/week recommended by UK guidelines [21].

Measures for secondary illnesses known to affect hearing were included. Hypertension was characterised as an average measurement of >140mm Hg systolic or >90mm Hg diastolic as per Agrawal et al. [6]. Diabetes [6] and stroke [22] were based on a diagnosis from a doctor. Cardiovascular disease [6] was indicated by self-reported heart attack, stroke, angina, deep vein thrombosis or other related condition. The use of medication for secondary conditions that may have ototoxic side effects [23] was also included.

The prevalence of disabling hearing loss is lower in high-income countries than elsewhere, partly due to hearing loss acquired during childhood [24]. As such, early life exposure (age <15) to the most common childhood diseases known to affect hearing were included in the model: meningitis [25], measles, mumps and rubella [26].

Correction for bias

Language proficiency was evaluated according to two-language based questions included in the UK Biobank's Fluid Intelligence (FI) assessment: *1) "Stop means the same as?" (Pause/Close/Cease/Break/Rest); 2) "Bud is to Flower as Child is to?" (Grow/Develop/Improve/Adult/Old).* Given the association between hearing loss and cognitive performance [27], numerical questions from the FI test were also included to correct for general cognitive ability. Respondents who did not take the cognitive tests, or ran out of time answering them, were categorised separately.

UK Biobank coded participants' ethnicity according to the 2001 Census ethnic groups. The present study was not concerned with differences between specific ethnic groups, but rather the risk that

For peer review only - http://bmjopen.bmj.com/site/about/guidelines.xhtml

non-native English speakers may be penalised on the DTT. As such, participants were categorised according to likely language proficiency. Those who migrated to the UK aged under 11 years were said to have native proficiency, as per Nazroo's investigation of variations in mental illness rates according to language ability [28]. Given the focus on language, the "White Other" ethnic group was classified as an ethnic minority group, with the exception of those who were born in a majority English-speaking country[1]. Ethnicity was ultimately coded as i) White, native English speaking (NE); ii) Black and Minority Ethnic (BME), native English speaking or early-migrating (NEEM) and iii) BME later-migrating.

The test centres used in Swansea and Wrexham were mobile units [29]. Anecdotal evidence suggested that acoustic background noise conditions were worse in these centres. Descriptive statistics (see supplementary table 7) revealed poorer average speech recognition thresholds for these centres. As such, we corrected for test centre type

Analysis

A Latent Factor Analysis (LFA) was initially used to combine all four hearing health measures into a single latent dimension. This model exhibited unusual behaviour by way of inflated coefficients for factor variables. This unreliability suggested that latent hearing health was not well-characterised by a single dimension; that is, self-reported hearing difficulties may not always correlate well with objective measures of hearing acuity. This inconsistency could also have been due to external factors that act differently upon each measure, e.g. propensity to report health problems, or bias in the hearing test for certain groups.

Latent Class Analysis (LCA) was employed to overcome the constraints of an LFA by allowing for the existence of arbitrarily different "classes" of latent hearing health. A latent construct of hearing health was formed using the four hearing measures. The suitability of this construct as a proxy to understand the latent trait of hearing health was evaluated by studying its association with known correlates of hearing loss. Analyses were performed in MPlus Version 8 using the three-step approach to correct for classification uncertainty in the latent classes [30].

Patient and public involvement

Although UK Biobank participants were involved in the design of the biobank resource itself, neither they, or any member of the public, were involved in this secondary analysis.

[1] Australia, Canada, Channel Islands, Ireland, Isle of Man, New Zealand, UK, USA

For peer review only - http://bmjopen.bmj.com/site/about/guidelines.xhtml

Table 1: Sample characteristics (N=151,271). Values are number (percentage).

	White, native English speaking (NE)	BME, native English speaking or early-migrating (NEEM)	BME later-migrating
**N**	**134,566**	**5,942**	**10,763**
**Age**			
40-44	12,634 (9.4)	1,399 (23.5)	1,751 (16.3)
45-49	15,811 (11.7)	1,534 (25.8)	1,650 (15.3)
50-54	19,136 (14.2)	1,249 (21.0)	1,776 (16.5)
55-59	23,408 (17.4)	798 (13.4)	1,914 (17.8)
60-64	35,446 (26.3)	606 (10.2)	2,010 (18.7)
65+	28,131 (20.9)	356 (6.0)	1,662 (15.4)
**Sex**			
Female	72,163 (53.6)	3,382 (56.9)	6,091 (56.6)
Male	62,403 (46.4)	2,560 (43.1)	4,672 (43.4)
**Townsend deprivation (mean)**	-1.4	0.3	0.9
**Educational qualifications**			
CSE/GCSE/A-Level	31,004 (23.0)	1,413 (23.8)	2,003 (18.6)
Degree/Higher/Prof qual	82,372 (61.2)	4,043 (68.0)	7,202 (66.9)
No formal qualifications	21,190 (15.7)	486 (8.2)	1,558 (14.5)
**Ethnic group**			
White British or Irish	133,811 (99.4)	0 (0.0)	0 (0.0)
White Other	755 (0.6)	1,915 (32.2)	2,943 (27.3)
Asian Bangladeshi	0 (0.0)	20 (0.3)	46 (0.4)
Asian Indian	0 (0.0)	824 (13.9)	2,216 (20.6)
Asian Other	0 (0.0)	93 (1.6)	739 (6.9)
Asian Pakistani	0 (0.0)	206 (3.5)	392 (3.6)
Chinese	0 (0.0)	70 (1.2)	471 (4.4)
Black African	0 (0.0)	197 (3.3)	1,284 (11.9)
Black Caribbean	0 (0.0)	1,396 (23.5)	951 (8.8)
Black Other	0 (0.0)	22 (0.4)	30 (0.3)
Mixed	0 (0.0)	816 (13.7)	318 (3.0)
Other	0 (0.0)	383 (6.4)	1,373 (12.8)
**Hearing measures**			
DTT: 'Normal'	119,260 (88.6)	5,331 (89.7)	7,251 (67.4)
DTT: 'Insufficient' or 'Poor'	15,306 (11.4)	611 (10.3)	3,512 (32.6)
Does not use hearing aid	130,507 (97.0)	5,866 (98.7)	10,566 (98.2)
Uses hearing aid	4,059 (3.0)	76 (1.3)	197 (1.8)
No self-reported hearing diff.	96,401 (71.6)	4,779 (80.4)	8,822 (82.0)
Self-reported hearing diff.	38,165 (28.4)	1,163 (19.6)	1,941 (18.0)
No self-reported h/diff. in noise	85,758 (63.7)	4,274 (71.9)	7,362 (68.4)
Self-reported h/diff. in noise	48,808 (36.3)	1,668 (28.1)	3,401 (31.6)

** Mixed group includes "White and Black Caribbean", "White and Black African", "White and Asian" and "Other Mixed"; Other group includes "Arab" and "Any other ethnic groups"*

For peer review only - http://bmjopen.bmj.com/site/about/guidelines.xhtml

RESULTS

Sample characteristics

165,309 participants provided valid hearing test data, reducing to 151,271 after the removal of cases having missing data (Supplementary figure 2). Table 1 shows the characteristics of the sample for each group in the analysis. Levels of 'poor' or 'insufficient' hearing according to the DTT are similar for the White NE and BME NEEM group; however, the later-migrating BME group had over twice the levels of hearing loss according to DTT performance. The two BME groups had similar levels of self-reported hearing difficulty.

Latent variable analysis

A combination of exploratory and confirmatory approaches was used to determine the properties of the hearing health latent classifications. It was expected that there would be a particular group who exhibited poor performance on the DTT, but did not self-report hearing problems or use a hearing aid. To test this, 3-class and 4-class confirmatory models were created with this group specified; other group thresholds were unconstrained, allowing the model to determine their properties. However, this class did not fit well with the data, having very low membership in both models.

Accordingly, an exploratory approach was revisited: unconstrained models with 2 to 7 classes were created, and the model with best fit was selected. A mixture of Bayesian Information Criterion (BIC) and Bootstrapped Likelihood Ratio Test (BLRT) is recommended for determining the optimum number of classes in a latent class analysis [31]; however, the BLRT models for 4 vs 3 classes were not reliable due to a non-positive definite first-order derivative product matrix; as such, the BIC alone was used to determine this number (Supplementary table 3).

Figure 1: Probabilities of 3-class LCA model

For peer review only

For peer review only - http://bmjopen.bmj.com/site/about/guidelines.xhtml

Hearing health classes

Figure 1 illustrates the hearing health classes produced in the 3-class model, which were interpreted as follows:

'Normal' (prevalence 60.4%): characterised by low levels of hearing aid use, poor/insufficient hearing on the DTT or reporting hearing difficulty more generally.

'Generally poor' (prevalence 6.9%): those who reported hearing difficulties, the highest levels of poor/insufficient DTT performance and used hearing aids.

'Only subjectively poor' (prevalence 32.7%): those who reported hearing difficulties but had a low probability of having poor/insufficient hearing on the DTT.

Latent class regression models

A multinomial logistic regression was performed using class membership as the dependent variable. The latent classes were regressed on a range of demographic, socioeconomic and health-related factors, as well as known biases in the dataset. Correlates were introduced hierarchically (see supplementary tables 4-6) to evaluate whether the ethnic inequalities could be explained by the introduction of particular factors. However, the effects of ethnicity remained fairly constant across model iterations.

Uncertainty in the latent class classification was compensated for using the manual "three step" procedure [30]. To test for bias in the DTT, the latent class regression models were expanded by additionally regressing the observed DTT outcome directly on variables hypothesised to be related to language skills (ethnicity/migration history, language test score and numerical test score) and known biases (whether tested in a mobile centre). A non-zero estimate for these direct effects would indicate bias (or "differential item functioning" as it is referred to in psychometrics). Table 2 shows results for these key variables before and after these direct effects were introduced.

Without the DTT direct effects, the BME NEEM group had significantly lower odds (OR 0.67, 95% CI 0.53-0.86) of being in the Generally-Poor compared to the normal hearing group; however, these odds were no longer significant (OR 0.83, 95% CI 0.68-1.03) once direct effects were introduced. Higher numeric and language scores caused a significant reduction in the likelihood of having poor or insufficient hearing on the DTT, but did not have a significant impact on group membership. The later-migrating BME group were much more likely than the White NE group to have insufficient or poor hearing (OR 3.85, 95% CI 3.64-4.07).

For peer review only

Table 2: Coefficients and 95% CI of key variables from multinomial logistic regression

Predictor	Latent hearing health class: Only Subjectively Poor OR (95% CI)	Generally Poor OR (95% CI)	Normal (ref) OR (95% CI)
Excluding DTT direct effects			
BME (NEEM)	0.86 (0.80 to 0.93)***	0.67 (0.53 to 0.86)**	1.00
BME, later-migrating	0.94 (0.89 to 1.00)	0.52 (0.44 to 0.62)***	1.00
Language score: 1 correct	1.01 (0.95 to 1.08)	0.92 (0.81 to 1.05)	1.00
Language score: 2 correct	1.05 (1.00 to 1.10)	0.85 (0.77 to 0.95)**	1.00
Language score: Not taken	0.91 (0.81 to 1.03)	0.86 (0.67 to 1.11)	1.00
Numeric score: >50% correct	1.04 (1.00 to 1.09)	1.02 (0.93 to 1.13)	1.00
Numeric score: Not taken	1.05 (0.94 to 1.17)	1.22 (0.97 to 1.53)	1.00
Mobile test centre	0.98 (0.88 to 1.09)	1.05 (0.83 to 1.32)	1.00
Including DTT direct effects			
BME (NEEM)	0.86 (0.80 to 0.93)***	0.83 (0.68 to 1.03)	1.00
BME, later-migrating	0.94 (0.89 to 1.00)	0.57 (0.49 to 0.67)***	1.00
Language score: 1 correct	1.00 (0.94 to 1.06)	0.98 (0.87 to 1.10)	1.00
Language score: 2 correct	1.03 (0.98 to 1.09)	0.91 (0.83 to 1.00)	1.00
Language score: Not taken	0.89 (0.79 to 1.00)	0.94 (0.76 to 1.17)	1.00
Numeric score: >50% correct	1.04 (0.99 to 1.09)	1.05 (0.96 to 1.15)	1.00
Numeric score: Not taken	1.08 (0.97 to 1.20)	1.10 (0.90 to 1.35)	1.00
Mobile test centre	1.00 (0.90 to 1.12)	0.94 (0.76 to 1.17)	1.00

DTT direct effects	OR of having insufficient or poor hearing
BME (NEEM)	1.04 (0.95 to 1.15)
BME, later-migrating	3.85 (3.64 to 4.07)***
Language score: 1 correct	0.69 (0.65 to 0.74)***
Language score: 2 correct	0.61 (0.58 to 0.65)***
Language score: Not taken	1.12 (1.00 to 1.26)*
Numeric score: >50% correct	0.71 (0.67 to 0.75)***
Numeric score: Not taken	1.34 (1.20 to 1.49)***
Mobile test centre	5.52 (4.99 to 6.10)***

All models controlled for age, sex, education, deprivation score, noise exposure, use of ototoxic medication, medical history, smoking and alcohol use, childhood illnesses and evaluation at a mobile test centre. Asterisk denotes significance: * 95% interval ** 99% interval *** 99.9% interval

For peer review only - http://bmjopen.bmj.com/site/about/guidelines.xhtml

DISCUSSION

This study further investigated the ethnic differences in hearing health observed by Dawes et al. [4] by considering the heterogeneity in language ability of the ethnic minority group, and by validating the results of the DTT against other hearing health outcomes.

By dividing the ethnic minority cohort according to migration age, the present research reveals intergenerational differences in DTT outcomes. As hypothesised, it is the later-migrating BME ethnic minority group who bear the burden of poorer hearing outcomes: the UK-born/early-migrating BME cohort had DTT results that were indistinguishable from the White NE cohort.

Aside from the DTT, the LFA was constructed using self-reported measures, which are thought to be reliable measures of hearing health. This construct indicates that ethnic minority groups are most likely to have normal hearing. However, even after correcting for hypothesised sources of bias, the results from the DTT suggest poorer hearing outcomes for the later-migrating BME group. This is consistent with literature showing ethnic inequalities in other physical and mental health outcomes [3], which have been shown to have multiple causes, including socioeconomic and demographic factors, as well as stress caused by experiences of racial harassment and discrimination [32].

A secondary finding of this study regards mobile test centres, which caused the largest bias in the DTT, possibly due to elevated background noise levels in the test centres. Respondents who were tested at these centres were 5.52 times (95% CI 4.99-6.10) more likely to have poor/insufficient hearing on the DTT, but were not more likely to have any particular hearing classification. It is also of note that those who did not take the cognitive tests had worse DTT scores. It may be that a cognitive or language difficulty precluded participants from taking, or finishing, these tests.

The LFA produced classes that were substantively consistent with the literature. The likelihood of being in the Generally-Poor hearing group was increased by exposure to noise and most of the health conditions known to affect hearing. The strongest indicator for the Only Subjectively-Poor hearing group was exposure to noise, which seems consistent with a cohort who are concerned about their hearing. Although it may seem unusual that this cohort would perform well on a speech recognition test yet report hearing difficulties in daily life, such phenomena are not uncommon in the literature; explanations include audiometric notches [33], cochlear synaptopathy [34], and health factors unique to the individual [35].

One limitation to this study regards language measures: proficiency was determined by responses to just two questions, so may be imprecise. Despite this, the measures are still able to provide evidence for the hypothesis of DTT bias, as the LFA outcomes did not exhibit any dependency on language. It must also be noted that the BME classes contain the "White Other" group, which may include participants from Western Europe who are not generally designated as minority ethnic.

For peer review only - http://bmjopen.bmj.com/site/about/guidelines.xhtml

Regarding generalisability, the UK Biobank, while not employing a representative sample, is thought to be generalisable for disease-exposure relationships [36].

The authors of the Biobank study did not explicitly state why the DTT was used over the Pure-tone Average method, but the available information points to practical factors, such the "minimal impact on throughput" by incorporating the test with the existing touchscreen questionnaire [37], and no requirement for calibrated equipment or a sound-treated test booth. Unfortunately, use of the DTT may have resulted in a test that is biased for non-native English speakers.

Even after accounting for this bias, the results suggest that there are still ethnic inequalities in hearing health, albeit with contradiction between objective and subjective outcomes. Designers of future studies must take precautions to ensure that this inequality can be reliably analysed by ensuring that objective measures are not language-dependent. The present study may also have implications for hearing screening applications, which many countries currently implement using the DTT [38]. Digit Triplet Tests are being translated to an increasing number of languages [39], although it may be some time before tests are available in all 6,500 languages spoken globally. Until such time, non-linguistic hearing tests may be the most practical solution to lessen the dependency on language proficiency in objective measures of hearing.

For peer review only

FUNDING

This work was supported by the Economic and Social Research Council [grant number ES/P000665/1] and the NIHR Manchester Biomedical Research Centre.

ACKNOWLEDGEMENTS

We used the STROBE cross sectional checklist when writing our report [40].

For peer review only - http://bmjopen.bmj.com/site/about/guidelines.xhtml

REFERENCES

1 Vos T, Allen C, Arora M, *et al.* Global, regional, and national incidence, prevalence, and years lived with disability for 310 diseases and injuries, 1990–2015: a systematic analysis for the Global Burden of Disease Study 2015. *Lancet* 2016;**388**:1545–602. doi:10.1016/S0140-6736(16)31678-6

2 Action on Hearing Loss. Facts and figures on hearing loss, deafness and tinnitus, A1205/0416. 2016.

3 Department of Health. Tackling health inequalities: 10 years on a review of developments in tackling health inequalities in England over the last 10 years. 2009.

4 Dawes P, Fortnum H, Moore DR, *et al.* Hearing in middle age: a population snapshot of 40–69 year olds in the UK. *Ear Hear* 2014;**35**:e44–51. doi:10.1097/AUD.0000000000000010

5 Bécares L. Which ethnic groups have the poorest health? Ethnic health inequalities 1991 to 2011. 2013. http://www.ethnicity.ac.uk/medialibrary/briefingsupdated/which-ethnic-groups-have-the-poorest-health.pdf (accessed 4 Sep 2017).

6 Agrawal Y, Platz EA, Niparko JK. Prevalence of Hearing Loss and Differences by Demographic Characteristics Among US Adults: Data From the National Health and Nutrition Examination Survey, 1999-2004. *Arch Intern Med* 2008;**168**:1522. doi:10.1001/archinte.168.14.1522

7 NatCen. Nurse project instructions P8814. 2014. http://doc.ukdataservice.ac.uk/doc/7919/mrdoc/pdf/7919_hse2014_supporting_documents.pdf

8 NatCen. ELSA Wave 7 Mainstage Interviewer Project Instructions. 2014.

9 Jansen S, Luts H, Wagener KC, *et al.* The French digit triplet test: a hearing screening tool for speech intelligibility in noise. *Int J Audiol* 2010;**49**:378–87. doi:10.3109/14992020903431272

10 Mayo LH, Florentine M, Buus S. Age of second-language acquisition and perception of speech in noise. *J Speech Lang Hear Res* 1997;**40**:686–93.http://www.ncbi.nlm.nih.gov/pubmed/9210123 (accessed 18 Mar 2018).

11 Warzybok A, Wagener KC, Brand T, *et al.* Influence of the linguistic complexity in relation to speech material on non-native speech perception in noise. In: *DAGA 2010 - Berlin*. 2010.

12 Allen N, Sudlow C, Downey P, *et al.* UK Biobank: Current status and what it means for epidemiology. *Heal Policy Technol* 2012;**1**:123–6. doi:10.1016/j.hlpt.2012.07.003

13 Smits C, Kapteyn TS, Houtgast T. Development and validation of an automatic speech-in-noise screening test by telephone. *Int J Audiol* 2004;**43**:15–28.http://www.ncbi.nlm.nih.gov/pubmed/14974624 (accessed 6 Jul 2017).

14 Kramer SE, Kapteyn TS, Festen JM, *et al.* The self-reported handicapping effect of hearing disabilities. *Int J Audiol* Published Online First: 1998. doi:10.3109/00206099809072984

15 Hall SJ. *The Development of a New English Sentence In Noise Test and an English Number Recognition Test*. 2006.

16 Corso JF. Age and sex differences in pure-tone thresholds. Survey of hearing levels from 18 to 65 years. *Arch Otolaryngol* Published Online First: 1963. doi:10.1001/archotol.1963.00750010399008

17 Baraldi GDS, De Almeida LC, Borges ACDC. Hearing loss in aging. *Braz J Otorhinolaryngol* 2007;**73**:64–70. doi:10.1016/s1808-8694(15)31123-x

18 Rigters SC, Metselaar M, Wieringa MH, *et al.* Contributing Determinants to Hearing Loss in Elderly Men and

For peer review only - http://bmjopen.bmj.com/site/about/guidelines.xhtml

Women: Results from the Population-Based Rotterdam Study. *Audiol Neurotol* 2016;**21**:10–5. doi:10.1159/000448348

19 Khil L, Wellmann J, Berger K. Determinants of Single and Multiple Sensory Impairments in an Urban Population. *Otolaryngol Neck Surg* 2015;**153**:364–71. doi:10.1177/0194599815588913

20 Popelka MM, Cruickshanks KJ, Wiley TL, *et al.* Moderate alcohol consumption and hearing loss: a protective effect. *J Am Geriatr Soc* 2000;**48**:1273–8.http://www.ncbi.nlm.nih.gov/pubmed/11037015

21 Department of Health. UK Chief Medical Officers' Low Risk Drinking Guidelines. 2016. https://www.gov.uk/government/uploads/system/uploads/attachment_data/file/545937/UK_CMOs__report.pdf (accessed 27 Aug 2017).

22 Kuo C-L, Shiao A-S, Wang S-J, *et al.* Risk of sudden sensorineural hearing loss in stroke patients: A 5-year nationwide investigation of 44,460 patients. *Medicine (Baltimore)* 2016;**95**:e4841. doi:10.1097/MD.0000000000004841

23 Schacht J, Talaska AE, Rybak LP. Cisplatin and aminoglycoside antibiotics: hearing loss and its prevention. *Anat Rec (Hoboken)* 2012;**295**:1837–50. doi:10.1002/ar.22578

24 Duthey B. *Background Paper 6.21: Hearing Loss.* 2013.https://www.who.int/medicines/areas/priority_medicines/BP6_21Hearing.pdf

25 Fortnum HM. Hearing impairment after bacterial meningitis: A review. Arch. Dis. Child. 1992. doi:10.1136/adc.67.9.1128

26 Cohen BE, Durstenfeld A, Roehm PC. Viral causes of hearing loss: A review for hearing health professionals. *Trends Hear* Published Online First: 2014. doi:10.1177/2331216514541361

27 Yuan J, Sun Y, Sang S, *et al.* The risk of cognitive impairment associated with hearing function in older adults: A pooled analysis of data from eleven studies. *Sci Rep* 2018;**8**:1–10. doi:10.1038/s41598-018-20496-w

28 Nazroo JY. *Ethnicity And Mental Health: Findings From A National Community Survey.* Policy Studies Institute 1999.

29 UK Biobank EGC. Minutes of UK Biobank Ethics and Governance Council Twenty Third Meeting. 2010. https://egcukbiobank.org.uk/sites/default/files/meetings/EGC23 agenda and report.pdf

30 Asparouhov T, Muthén B. Auxiliary Variables in Mixture Modeling: Three-Step Approaches Using Mplus. *Struct Equ Model* Published Online First: 2014. doi:10.1080/10705511.2014.915181

31 Nylund KL, Asparouhov T, Muthén BO. Deciding on the number of classes in latent class analysis and growth mixture modeling: A Monte Carlo simulation study. *Struct Equ Model* Published Online First: 2007. doi:10.1080/10705510701575396

32 Nazroo JY. The structuring of ethnic inequalities in health: economic position, racial discrimination, and racism. *Am J Public Health* 2003;**93**:277–84.http://www.ncbi.nlm.nih.gov/pubmed/12554585 (accessed 27 Jun 2017).

33 Zhao F, Stephens D. Audioscan testing in patients with King-Kopetzky syndrome. *Acta Otolaryngol* Published Online First: 1999. doi:10.1080/00016489950181297

34 Bharadwaj HM, Masud S, Mehraei G, *et al.* Individual differences reveal correlates of hidden hearing deficits. *J Neurosci* Published Online First: 2015. doi:10.1523/JNEUROSCI.3915-14.2015

35 Pronk M, Deeg DJH, Kramer SE. Explaining discrepancies between the digit triplet speech-in-noise test score and self-reported hearing problems in older adults. *J Speech, Lang Hear Res* Published Online First: 2018.

For peer review only - http://bmjopen.bmj.com/site/about/guidelines.xhtml

doi:10.1044/2018_JSLHR-H-17-0124

36 Fry A, Littlejohns TJ, Sudlow C, *et al.* Comparison of Sociodemographic and Health-Related Characteristics of UK Biobank Participants with the General Population. *Am J Epidemiol* Published Online First: 21 June 2017. doi:10.1093/aje/kwx246

37 UK Biobank. UK Biobank Ethics and Governance Council Report - Fifteenth Meeting. 2008.

38 Watson CS, Kidd GR, Miller JD, *et al.* Telephone screening tests for functionally impaired hearing: Current use in seven countries and development of a US version. *J Am Acad Audiol* Published Online First: 2012. doi:10.3766/jaaa.23.10.2

39 Zokoll MA, Hochmuth S, Warzybok A, *et al.* Speech-in-noise tests for multilingual hearing screening and diagnostics. *Am J Audiol* Published Online First: 2013. doi:10.1044/1059-0889(2013/12-0061)

40 von Elm E, Altman DG, Egger M, *et al.* The strengthening the reporting of observational studies in epidemiology (STROBE) statement: Guidelines for reporting observational studies. *Int J Surg* Published Online First: 2014. doi:10.1016/j.ijsu.2014.07.013

For peer review only

SUPPLEMENTARY FIGURES

Figure 2: Derivation of final sample size

Table 3: BIC for exploratory latent class analysis (step 1)

Table 4: *Normal hearing* group Vs *Only Subjectively-Poor* hearing group

Table 5: *Normal hearing* group Vs *Generally-Poor hearing group*

Table 6: *DTT pathway*

Table 7: *SRT variation by test centre*

For peer review only

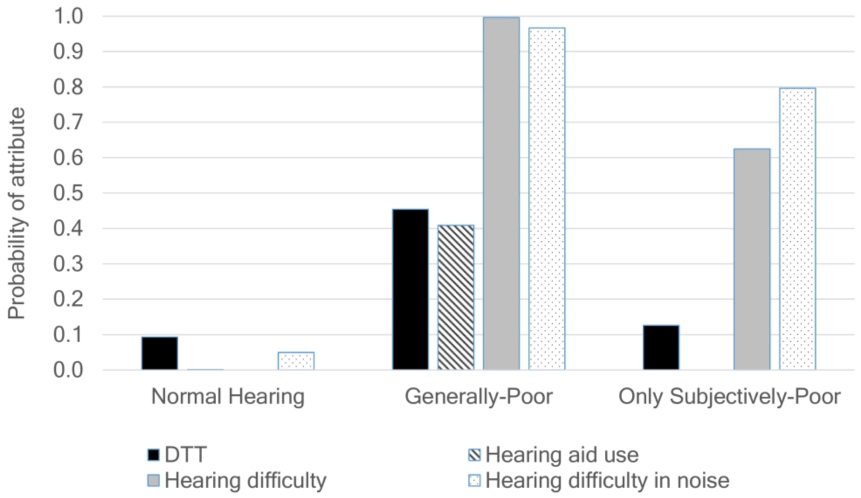

Figure 1: Probabilities of 3-class LCA model

297x209mm (300 x 300 DPI)

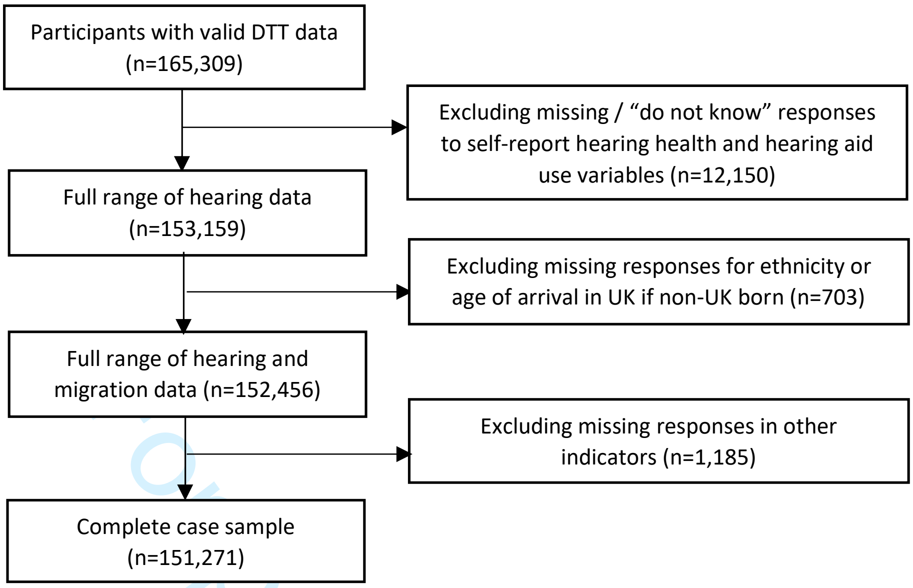

For peer review only

No. classes	BIC
2	456,798
3	455,476
4	455,506
5	455,565
6	455,625
7	455,684

For peer review only

For peer review only

	m1	m2	m3	m4	m5
**Covariate**	**Est**	**Est**	**Est**	**Est**	**Est**
Age	1.03 (1.03 to 1.03)	1.03 (1.03 to 1.03)	1.04 (1.04 to 1.04)	1.04 (1.04 to 1.04)	1.03 (1.03 to 1.04)
Sex: Male	1.71 (1.66 to 1.75)	1.69 (1.64 to 1.74)	1.36 (1.32 to 1.41)	1.36 (1.32 to 1.41)	1.37 (1.33 to 1.41)
BME (NEEM)	0.88 (0.82 to 0.95)	0.86 (0.80 to 0.92)	0.86 (0.80 to 0.92)	0.86 (0.80 to 0.93)	0.84 (0.78 to 0.91)
BME, later-migrating	0.90 (0.85 to 0.95)	0.86 (0.81 to 0.91)	0.91 (0.86 to 0.97)	0.94 (0.89 to 1.00)	0.94 (0.88 to 1.00)
Mobile test centre	1.00 (0.90 to 1.12)	1.00 (0.90 to 1.11)	0.98 (0.88 to 1.09)	0.98 (0.88 to 1.09)	1.00 (0.90 to 1.12)
Education: High School		0.88 (0.84 to 0.93)	0.99 (0.94 to 1.04)	0.97 (0.92 to 1.02)	1.00 (0.95 to 1.05)
Education: Degree		0.99 (0.95 to 1.03)	1.13 (1.08 to 1.17)	1.10 (1.05 to 1.15)	1.14 (1.09 to 1.20)
Townsend dep. Score		1.02 (1.01 to 1.02)	1.00 (1.00 to 1.01)	1.01 (1.00 to 1.01)	1.00 (1.00 to 1.01)
Exposure to loud noise			1.84 (1.78 to 1.91)	1.85 (1.78 to 1.91)	1.85 (1.78 to 1.91)
Exposure to loud music			1.82 (1.74 to 1.89)	1.82 (1.75 to 1.89)	1.81 (1.74 to 1.89)
Use of ototoxic meds.			1.05 (1.01 to 1.10)	1.05 (1.01 to 1.10)	1.06 (1.01 to 1.11)
Stroke			1.07 (0.94 to 1.21)	1.07 (0.94 to 1.22)	1.00 (0.87 to 1.14)
Diabetes			1.03 (0.96 to 1.10)	1.03 (0.97 to 1.10)	1.02 (0.95 to 1.09)
Cardiovascular illness			1.12 (1.06 to 1.19)	1.12 (1.06 to 1.19)	1.13 (1.07 to 1.19)
Hypertension			0.93 (0.90 to 0.96)	0.93 (0.90 to 0.96)	0.93 (0.90 to 0.95)
Smoker (at some stage)			1.11 (1.07 to 1.14)	1.11 (1.07 to 1.14)	1.11 (1.07 to 1.14)
Alcohol: Former			1.10 (1.01 to 1.20)	1.10 (1.01 to 1.20)	1.08 (0.99 to 1.18)
Alcohol: Meets guidelines			1.00 (0.95 to 1.05)	0.99 (0.94 to 1.04)	1.01 (0.96 to 1.06)
Alcohol: Exceeds g/l			1.02 (0.96 to 1.07)	1.01 (0.96 to 1.06)	1.02 (0.97 to 1.08)
Meningitis as child			1.62 (1.12 to 2.35)	1.62 (1.11 to 2.35)	1.62 (1.13 to 2.32)
MMR as child			1.02 (0.90 to 1.16)	1.02 (0.89 to 1.16)	0.98 (0.86 to 1.12)
Language score: 1 correct				1.01 (0.95 to 1.08)	1.00 (0.94 to 1.06)
Language score: 2 correct				1.05 (1.00 to 1.10)	1.03 (0.98 to 1.09)
Language score: Not taken				0.91 (0.81 to 1.03)	0.89 (0.79 to 1.00)
Numeric score: >50% correct				1.04 (1.00 to 1.09)	1.04 (0.99 to 1.09)
Numeric score: Not taken				1.05 (0.94 to 1.17)	1.08 (0.97 to 1.20)

For peer review only

Covariate	m1 Est	m2 Est	m3 Est	m4 Est	m5 Est
Age	1.13 (1.12 to 1.14)	1.13 (1.12 to 1.13)	1.13 (1.13 to 1.14)	1.13 (1.13 to 1.14)	1.15 (1.15 to 1.16)
Sex: Male	1.93 (1.82 to 2.06)	1.93 (1.81 to 2.06)	1.36 (1.27 to 1.46)	1.36 (1.26 to 1.46)	1.33 (1.25 to 1.42)
BME (NEEM)	0.74 (0.58 to 0.94)	0.72 (0.56 to 0.91)	0.68 (0.54 to 0.87)	0.67 (0.53 to 0.86)	0.83 (0.68 to 1.03)
BME, later-migrating	0.58 (0.50 to 0.68)	0.55 (0.47 to 0.65)	0.55 (0.47 to 0.65)	0.52 (0.44 to 0.62)	0.57 (0.49 to 0.67)
Mobile test centre	1.13 (0.90 to 1.41)	1.12 (0.90 to 1.40)	1.05 (0.84 to 1.32)	1.05 (0.83 to 1.32)	0.94 (0.76 to 1.17)
Education: High School		0.63 (0.57 to 0.69)	0.80 (0.72 to 0.88)	0.83 (0.75 to 0.91)	0.80 (0.73 to 0.87)
Education: Degree		0.71 (0.66 to 0.77)	0.90 (0.83 to 0.98)	0.94 (0.87 to 1.03)	0.86 (0.80 to 0.93)
Townsend dep. Score		1.03 (1.02 to 1.04)	1.01 (0.99 to 1.02)	1.01 (0.99 to 1.02)	1.02 (1.01 to 1.03)
Exposure to loud noise			2.89 (2.69 to 3.10)	2.86 (2.67 to 3.07)	2.77 (2.61 to 2.95)
Exposure to loud music			2.15 (1.95 to 2.37)	2.14 (1.94 to 2.36)	2.13 (1.95 to 2.32)
Use of ototoxic meds.			1.16 (1.06 to 1.27)	1.16 (1.06 to 1.27)	1.13 (1.05 to 1.22)
Stroke			1.15 (0.93 to 1.42)	1.14 (0.92 to 1.42)	1.33 (1.11 to 1.60)
Diabetes			1.21 (1.07 to 1.36)	1.20 (1.07 to 1.36)	1.24 (1.12 to 1.37)
Cardiovascular illness			1.24 (1.12 to 1.37)	1.24 (1.12 to 1.37)	1.20 (1.10 to 1.31)
Hypertension			0.93 (0.87 to 1.00)	0.93 (0.87 to 1.00)	0.95 (0.89 to 1.01)
Smoker (at some stage)			1.13 (1.06 to 1.21)	1.14 (1.06 to 1.21)	1.13 (1.07 to 1.20)
Alcohol: Former			1.04 (0.87 to 1.25)	1.05 (0.87 to 1.26)	1.14 (0.97 to 1.33)
Alcohol: Meets guidelines			1.01 (0.90 to 1.12)	1.01 (0.91 to 1.13)	0.95 (0.87 to 1.05)
Alcohol: Exceeds g/l			0.81 (0.72 to 0.91)	0.82 (0.73 to 0.92)	0.79 (0.72 to 0.88)
Meningitis as child			2.93 (1.59 to 5.41)	2.95 (1.60 to 5.43)	2.88 (1.64 to 5.08)
MMR as child			1.12 (0.84 to 1.49)	1.13 (0.84 to 1.51)	1.29 (1.01 to 1.65)
Language score: 1 correct				0.92 (0.81 to 1.05)	0.98 (0.87 to 1.10)
Language score: 2 correct				0.85 (0.77 to 0.95)	0.91 (0.83 to 1.00)
Language score: Not taken				0.86 (0.67 to 1.11)	0.94 (0.76 to 1.17)
Numeric score: >50% correct				1.02 (0.93 to 1.13)	1.05 (0.96 to 1.15)
Numeric score: Not taken				1.22 (0.97 to 1.53)	1.10 (0.90 to 1.35)

For peer review only

DTT pathway	m1 Est	m2 Est	m3 Est	m4 Est	m5 Est
BME, migrated age <12					1.04 (0.95 to 1.15)
BME, migrated age >= 12					3.85 (3.64 to 4.07)
Language score: 1 correct					0.69 (0.65 to 0.74)
Language score: 2 correct					0.61 (0.58 to 0.65)
Language score: Not taken					1.12 (1.00 to 1.26)
Numeric score: >50% correct					0.71 (0.67 to 0.75)
Numeric score: Not taken					1.34 (1.20 to 1.49)
Mobile test centre					5.52 (4.99 to 6.10)

For peer review only

Test centre	Average SRT Mean	Standard Deviation
Birmingham	-7.1	1.8
Bristol	-7.1	1.6
Croydon	-7.0	1.8
Hounslow	-7.4	1.9
Liverpool	-7.5	1.7
Middlesbrough	-7.5	1.9
Nottingham	-6.8	1.9
Sheffield	-7.7	1.6
Swansea	-6.0	1.8
Wrexham	-6.0	1.9

For peer review only

Reporting checklist for cross sectional study.

Based on the STROBE cross sectional guidelines.

Instructions to authors

Complete this checklist by entering the page numbers from your manuscript where readers will find each of the items listed below.

Your article may not currently address all the items on the checklist. Please modify your text to include the missing information. If you are certain that an item does not apply, please write "n/a" and provide a short explanation.

Upload your completed checklist as an extra file when you submit to a journal.

In your methods section, say that you used the STROBE cross sectionalreporting guidelines, and cite them as:

von Elm E, Altman DG, Egger M, Pocock SJ, Gotzsche PC, Vandenbroucke JP. The Strengthening the Reporting of Observational Studies in Epidemiology (STROBE) Statement: guidelines for reporting observational studies.

For peer review only

		Reporting Item	Page Number
**Title and abstract**			
Title	#1a	Indicate the study's design with a commonly used term in the title or the abstract	2
Abstract	#1b	Provide in the abstract an informative and balanced summary	2

		of what was done and what was found	
**Introduction**			
Background / rationale	#2	Explain the scientific background and rationale for the investigation being reported	3
Objectives	#3	State specific objectives, including any prespecified hypotheses	3-4
**Methods**			
Study design	#4	Present key elements of study design early in the paper	4
Setting	#5	Describe the setting, locations, and relevant dates, including periods of recruitment, exposure, follow-up, and data collection	4
Eligibility criteria	#6a	Give the eligibility criteria, and the sources and methods of selection of participants.	4
	#7	Clearly define all outcomes, exposures, predictors, potential confounders, and effect modifiers. Give diagnostic criteria, if applicable	4-6
Data sources / measurement	#8	For each variable of interest give sources of data and details of methods of assessment (measurement). Describe comparability of assessment methods if there is more than one group. Give information separately for for exposed and unexposed groups if applicable.	4-6
Bias	#9	Describe any efforts to address potential sources of bias	5-6

For peer review only

Study size	#10	Explain how the study size was arrived at	8
Quantitative variables	#11	Explain how quantitative variables were handled in the analyses. If applicable, describe which groupings were chosen, and why	4-6
Statistical methods	#12a	Describe all statistical methods, including those used to control for confounding	6
Statistical methods	#12b	Describe any methods used to examine subgroups and interactions	N/A
Statistical methods	#12c	Explain how missing data were addressed	8
Statistical methods	#12d	If applicable, describe analytical methods taking account of sampling strategy	N/A
Statistical methods	#12e	Describe any sensitivity analyses	N/A
**Results**			
Participants	#13a	Report numbers of individuals at each stage of study—eg numbers potentially eligible, examined for eligibility, confirmed eligible, included in the study, completing follow-up, and analysed. Give information separately for for exposed and unexposed groups if applicable.	8 (supp fig. 1)
Participants	#13b	Give reasons for non-participation at each stage	8 (supp fig. 1)

For peer review only

Participants	#13c	Consider use of a flow diagram	8 (supp fig. 1)
Descriptive data	#14a	Give characteristics of study participants (eg demographic, clinical, social) and information on exposures and potential confounders. Give information separately for exposed and unexposed groups if applicable.	7
Descriptive data	#14b	Indicate number of participants with missing data for each variable of interest	N/A
Outcome data	#15	Report numbers of outcome events or summary measures. Give information separately for exposed and unexposed groups if applicable.	N/A
Main results	#16a	Give unadjusted estimates and, if applicable, confounder-adjusted estimates and their precision (eg, 95% confidence interval). Make clear which confounders were adjusted for and why they were included	8-10 (supp. tbl. 4-6)
Main results	#16b	Report category boundaries when continuous variables were categorized	N/A
Main results	#16c	If relevant, consider translating estimates of relative risk into absolute risk for a meaningful time period	N/A
Other analyses	#17	Report other analyses done—e.g., analyses of subgroups and interactions, and sensitivity analyses	N/A

Discussion

For peer review only

Key results	#18	Summarise key results with reference to study objectives	11-12
Limitations	#19	Discuss limitations of the study, taking into account sources of potential bias or imprecision. Discuss both direction and magnitude of any potential bias.	11-12
Interpretation	#20	Give a cautious overall interpretation considering objectives, limitations, multiplicity of analyses, results from similar studies, and other relevant evidence.	11-12
Generalisability	#21	Discuss the generalisability (external validity) of the study results	12
**Other Information**			
Funding	#22	Give the source of funding and the role of the funders for the present study and, if applicable, for the original study on which the present article is based	12

Notes:

- 13a: 8 (supp fig. 1)
- 13b: 8 (supp fig. 1)
- 13c: 8 (supp fig. 1) The STROBE checklist is distributed under the terms of the Creative Commons Attribution License CC-BY. This checklist was completed on 22. February 2020 using https://www.goodreports.org/, a tool made by the EQUATOR Network in collaboration with Penelope.ai

For peer review only

BMJ Open

Understanding ethnic inequalities in hearing health in the UK: a cross-sectional study of the link between language proficiency and performance on the Digit Triplet Test

Journal:	*BMJ Open*
Manuscript ID	bmjopen-2020-042571.R1
Article Type:	Original research
Date Submitted by the Author:	18-Nov-2020
Complete List of Authors:	Taylor, Harry; The University of Manchester School of Social Sciences, Social Statistics; The University of Manchester Cathie Marsh Institute for Social Research<br>Shryane, Nick; The University of Manchester School of Social Sciences, Social Statistics; The University of Manchester Cathie Marsh Institute for Social Research<br>Kapadia, Dharmi; The University of Manchester School of Social Sciences, Sociology; The University of Manchester Cathie Marsh Institute for Social Research<br>Dawes, Piers; The University of Manchester School of Health Sciences, Manchester Centre for Audiology and Deafness; Manchester University NHS Foundation Trust, Manchester Academic Health Science Centre<br>Norman, Paul; University of Leeds, School of Geography
**Primary Subject Heading**:	Epidemiology
Secondary Subject Heading:	Ear, nose and throat/otolaryngology, Public health
Keywords:	EPIDEMIOLOGY, Audiology < OTOLARYNGOLOGY, STATISTICS & RESEARCH METHODS

SCHOLARONE™
Manuscripts

I, the Submitting Author has the right to grant and does grant on behalf of all authors of the Work (as defined in the below author licence), an exclusive licence and/or a non-exclusive licence for contributions from authors who are: i) UK Crown employees; ii) where BMJ has agreed a CC-BY licence shall apply, and/or iii) in accordance with the terms applicable for US Federal Government officers or employees acting as part of their official duties; on a worldwide, perpetual, irrevocable, royalty-free basis to BMJ Publishing Group Ltd ("BMJ") its licensees and where the relevant Journal is co-owned by BMJ to the co-owners of the Journal, to publish the Work in this journal and any other BMJ products and to exploit all rights, as set out in our licence.

The Submitting Author accepts and understands that any supply made under these terms is made by BMJ to the Submitting Author unless you are acting as an employee on behalf of your employer or a postgraduate student of an affiliated institution which is paying any applicable article publishing charge ("APC") for Open Access articles. Where the Submitting Author wishes to make the Work available on an Open Access basis (and intends to pay the relevant APC), the terms of reuse of such Open Access shall be governed by a Creative Commons licence – details of these licences and which Creative Commons licence will apply to this Work are set out in our licence referred to above.

Other than as permitted in any relevant BMJ Author's Self Archiving Policies, I confirm this Work has not been accepted for publication elsewhere, is not being considered for publication elsewhere and does not duplicate material already published. I confirm all authors consent to publication of this Work and authorise the granting of this licence.

For peer review only

Understanding ethnic inequalities in hearing health in the UK: a cross-sectional study of the link between language proficiency and performance on the Digit Triplet Test

Corresponding author:

Harry Taylor, Room G45, Humanities Bridgeford Street, Oxford Road, Manchester, M13 9PL. harry.taylor-2@postgrad.manchester.ac.uk, +44 (0)161 306 1340

Co-authors:

1. **Nick Shryane**, The Department of Social Statistics, The University of Manchester, Manchester, UK.
   also The Cathie Marsh Institute for Social Research (CMI), The University of Manchester, Manchester, UK.
2. **Dharmi Kapadia**, The Cathie Marsh Institute for Social Research (CMI), The University of Manchester, Manchester, UK.
3. **Piers Dawes**, Manchester Centre for Audiology and Deafness, School of Health Sciences, University of Manchester, Manchester, UK;
   also Manchester University NHS Foundation Trust (MFT), Manchester Academic Health Science Centre, Manchester, UK;
   also Department of Linguistics, Macquarie University, Sydney, Australia;
4. **Paul Norman**, School of Geography, University of Leeds, Leeds, UK

For peer review only

ABSTRACT

Introduction

Research using UK Biobank data has shown ethnic inequalities in hearing health; however, the hearing test used may exhibit a disadvantage for non-native language speakers.

Objectives

To validate the results of the UK Biobank hearing test (Digit Triplet Test, DTT) against self-reported measures of hearing in the dataset and create classifications of hearing health. To observe if language proficiency and migration age have the same effect upon hearing health classification as upon the DTT in isolation. Our hypothesis is that language proficiency acts differently upon the DTT, demonstrating that the DTT is biased for non-native speakers of English.

Design

Latent classes representing profiles of hearing health were identified from the available hearing measures. Factors associated with class membership were tested using multinomial logistic regression models. Ethnicity was defined as i) White, native English-speaking, ii) ethnic minority, arrived in UK aged <12 or iii) ethnic minority, arrived aged =>12.

Participants

UK Biobank participants with valid hearing test results and associated covariates (N=151,268)

Outcome measures

DTT score, self-reported hearing difficulty, self-reported hearing difficulty in noise and hearing aid use

Results

Three classes of hearing health were found, 'Normal', 'Generally Poor' and 'Only Subjectively Poor'. In a model adjusting for known confounders of hearing loss, a poor or insufficient hearing test result was less likely for those with better language (OR 0.69, 0.65-0.74 95% CI) or numerical ability (OR 0.71, 0.67-0.75 95% CI) but more likely for those having migrated aged >= 12 (OR 3.85, 3.64-4.07 95% CI).

Conclusions

The DTT showed evidence of bias, having greater dependence upon language ability and migration age than other hearing indicators. Designers of future surveys and hearing screening applications may wish to consider the limitations of speech-in-noise tests in evaluating hearing acuity for populations that include non-native speakers.

For peer review only

ARTICLE SUMMARY

Strengths and limitations of this study

- To our knowledge, the first study comparing self-reported and psychophysical hearing outcomes for ethnic minorities using UK Biobank data
- Potential language bias in the UK Biobank's hearing test was investigated for the first time
- All available hearing outcomes from the UK Biobank were combined into 3 latent classes; the behaviour of these classes was substantively consistent with the literature
- This study uses the available language proficiency data in the UK Biobank, which is limited.

For peer review only

For peer review only - http://bmjopen.bmj.com/site/about/guidelines.xhtml

INTRODUCTION

Hearing impairment is one of the most common chronic age-related health conditions [1]; it is estimated that 1 in 5 people in the UK will have hearing loss, characterised by 25dB or more hearing loss in the better ear, by 2035 [2]. Ethnic inequalities in health outcomes are well-documented and have been a key focus for UK government over the last 25 years [3]. Yet research into ethnic inequalities in hearing health in the UK has been limited due to insufficient ethnic minority sample sizes.

This research study is concerned with the ethnic inequality observed by Dawes and colleagues while investigating the correlates of hearing loss using UK Biobank data [4]. The study found that people who reported a Non-White ethnic background were 3.27 (3.07-3.48, 95% CI) times more likely to have hearing loss than those who identified as White British. This result was consistent with reports of higher levels of other chronic health conditions among minority groups in the UK [5], but contradicted observations from the USA where hearing loss was 70% lower in Black groups compared to White groups [6].

Hearing acuity in the UK Biobank is measured using the Digit Triplet Test (DTT), a test of speech recognition that involves perception of three spoken digits. Other UK health surveys collect hearing data using methods based on pure-tone audiometry (PTA) [7,8], a non-language-based evaluation of hearing acuity employing detection of pure tones. The DTT generally shows good correlation with PTA measures [9]; however, the word-based approach of speech-in-noise tests measures language comprehension as well as hearing acuity. This could present a scenario whereby a poor test result for a non-native speaker is due not to poor hearing, but to a lower level of language proficiency compared with native speakers [10]. This language penalty has been shown to increase the more linguistically-complex the speech-in-noise test [11].

Given the potential conflation of language ability and hearing acuity in the DTT, the present study divides the ethnic minority cohort into those likely to have native-level English proficiency, and those who migrated to the UK as an adolescent or older. It is hypothesised that, correcting for respondents' self-reported hearing health and related factors, DTT scores for the older-migrating cohort will be worse due to their likely lower language proficiency.

The present research therefore aims to validate the results of the DTT against three self-reported measures of hearing provided in the UK Biobank. These measures will be used to create classifications of hearing health. We will see if language proficiency and migration age have the same effect upon the hearing health classifications as for the DTT in isolation.

Our hypothesis is that the DTT is biased for non-native speakers of English. If the DTT is not biased, it would be expected that language test scores would have the same effect across every measure of hearing in the dataset. A different outcome would indicate a dependency on language

For peer review only - http://bmjopen.bmj.com/site/about/guidelines.xhtml

ability. If, after controlling for language ability, the DTT still shows poorer outcomes for the ethnic minority groups, it can be concluded that inequalities are present regardless of any language bias.

METHOD

Sample and design

The study population was participants in the UK Biobank, a biomedical study of over 500,000 adults aged 40-69 recruited in the United Kingdom from 2006-2010 [12]. Participants were invited to the study based on their proximity to one of 25 test centres and being registered with the National Health Service [12]. The study design was cross-sectional, using only the first wave of UK Biobank responses. The present research considers the 151,268 adults who produced valid DTT results and had a complete set of variables. UK Biobank received ethical approval from the National Health Service Research Ethics Service North West (11/NW/0382). Explicit consent for secondary analyses such as the one reported here, was obtained from participants in the UK Biobank. We used the STROBE cross sectional checklist when writing our report [13].

Measures

Hearing health

Four measures of hearing health were used to form the hearing health classifications: the DTT, two self-reported measures of hearing difficulty, and self-reported hearing aid use. All data, including the psychophysical measure, were collected in a self-administered manner via the touchscreen questionnaire undertaken in Biobank test centres.

As a speech-in-noise test, the DTT was devised to detect impaired speech intelligibility in noisy conditions [14], which is one of the most frequently-reported hearing difficulties associated with hearing loss [15]. The DTT was originally validated using participants whose first language was English [16]. The test produces a Speech Recognition Threshold (SRT) measured using signal to noise ratio in decibels (dB) [14], with lower scores representing better performance. The lowest score in the better ear was used in the present study, with scores above -5.5dB characterised as 'insufficient/poor' as per Dawes et al. [4]. Respondents who stated they were completely deaf or used a cochlear implant were excluded due to having no DTT data.

For the self-reported measures, respondents were asked: "Do you have any difficulty with your hearing?" (*Yes / No / I am completely deaf / Do not know / Prefer not to answer),* and "Do you find it difficult to follow a conversation if there is background noise (such as TV, radio, children playing)?" (*Yes / No / Do not know / Prefer not to answer).* The completely deaf category was amalgamated with the "Yes" category. Respondents were asked if they currently use a hearing aid "most of the time" *(Yes / No / Prefer not to answer).* Participants responding "Do not know" or "Prefer not to answer" to any of the hearing questions were excluded from the dataset.

For peer review only - http://bmjopen.bmj.com/site/about/guidelines.xhtml

Correlates of hearing loss

Hearing health is affected by a number of demographic, socioeconomic and lifestyle factors, as well as co-morbidity from associated diseases [6]. These factors were controlled for in the final analysis.

Hearing loss is more likely for males, and with increasing age [17,18]. Socioeconomic status has been shown to affect hearing, including education [19] and income [20]. Education was coded as either no formal qualification, secondary-school level qualifications or any further education. The Townsend index, an area-level deprivation measure, was used instead of household income, which had a high number of missing values.

Exposure to noise is a well-established factor in hearing loss [6]. Measures were included for whether the participant had ever worked in an environment where they had to shout to be heard, or if they had previously been exposed to loud music.

Lifestyle factors known to affect hearing were included. Smoking [6] was categorised as never smoker or former/current smoker. Alcohol consumption [21] was calculated based on estimated weekly intake per type of drink, and an ethanol content estimate for each type. Where an estimate was unavailable, a predicted value was assigned according to alcohol intake frequency. The resulting intake was categorised against the 112g/week recommended by UK guidelines [22].

Measures for secondary illnesses known to affect hearing were included. Hypertension was characterised as an average measurement of >140mm Hg systolic or >90mm Hg diastolic as per Agrawal et al. [6]. Diabetes [6] and stroke [23] were based on a diagnosis from a doctor. Cardiovascular disease [6] was indicated by self-reported heart attack, stroke, angina, deep vein thrombosis or other related condition. The use of medication for secondary conditions that may have ototoxic side effects [24] was also included.

The prevalence of disabling hearing loss is lower in high-income countries than elsewhere, partly due to hearing loss acquired during childhood [25]. As such, early life exposure (age <15) to the most common childhood diseases known to affect hearing were included in the model: meningitis [26], measles, mumps and rubella [27].

Correction for bias

Language proficiency was evaluated according to two-language based questions included in the UK Biobank's Fluid Intelligence (FI) assessment: *1) "Stop means the same as?" (Pause/Close/Cease/Break/Rest); 2) "Bud is to Flower as Child is to?" (Grow/Develop/Improve/Adult/Old).* Given the association between hearing loss and cognitive performance [28], numerical questions from the FI test were also included to correct for general cognitive ability. Respondents who did not take the cognitive tests, or ran out of time answering them, were categorised separately.

UK Biobank coded participants' ethnicity according to the 2001 Census ethnic groups. The present study was not concerned with differences between specific ethnic groups, but rather the risk that

For peer review only - http://bmjopen.bmj.com/site/about/guidelines.xhtml

non-native English speakers may be penalised on the DTT. As such, participants were categorised according to likely language proficiency. Those who migrated to the UK aged under 11 years were said to have native proficiency, as per Nazroo's investigation of variations in mental illness rates according to language ability [29]. Given the focus on language, the "White Other" ethnic group was classified as an ethnic minority group, with the exception of those who were born in a majority English-speaking country[1]. Ethnicity was ultimately coded as i) White, native English speaking (NE); ii) Black and Minority Ethnic (BME), native English speaking or early-migrating (NEEM) and iii) BME later-migrating.

The test centres used in Swansea and Wrexham were mobile units [30]. Anecdotal evidence suggested that acoustic background noise conditions were worse in these centres. Descriptive statistics (see supplementary table 1) revealed poorer average speech recognition thresholds for these centres. As such, we corrected for test centre type

Analysis

A Latent Factor Analysis (LFA) was initially used to combine all four hearing health measures into a single latent dimension. This model exhibited unusual behaviour by way of inflated coefficients for factor variables. This unreliability suggested that latent hearing health was not well-characterised by a single dimension; that is, self-reported hearing difficulties may not always correlate well with psychophysical measures of hearing acuity. This inconsistency could also have been due to external factors that act differently upon each measure, e.g. propensity to report health problems, or bias in the hearing test for certain groups.

Latent Class Analysis (LCA) was employed to overcome the constraints of an LFA by allowing for the existence of arbitrarily different "classes" of latent hearing health. A latent construct of hearing health was formed using the four hearing measures. The suitability of this construct as a proxy to understand the latent trait of hearing health was evaluated by studying its association with known correlates of hearing loss. Analyses were performed in MPlus Version 8 using the three-step approach to correct for classification uncertainty in the latent classes [31].

Patient and public involvement

Although UK Biobank participants were involved in the design of the biobank resource itself, neither they, or any member of the public, were involved in this secondary analysis.

[1] Australia, Canada, Channel Islands, Ireland, Isle of Man, New Zealand, UK, USA

For peer review only - http://bmjopen.bmj.com/site/about/guidelines.xhtml

Table 1: Sample characteristics (N=151,268). Values are number (percentage).

	White, native English speaking (NE)	BME, native English speaking or early-migrating (NEEM)	BME later-migrating
**N**	**134,563**	**5,942**	**10,763**
**Age**			
40-44	12,634 (9.4)	1,399 (23.5)	1,751 (16.3)
45-49	15,809 (11.7)	1,534 (25.8)	1,650 (15.3)
50-54	19,136 (14.2)	1,249 (21.0)	1,776 (16.5)
55-59	23,407 (17.4)	798 (13.4)	1,914 (17.8)
60-64	35,446 (26.3)	606 (10.2)	2,010 (18.7)
65+	28,131 (20.9)	356 (6.0)	1,662 (15.4)
**Sex**			
Female	72,161 (53.6)	3,382 (56.9)	6,091 (56.6)
Male	62,402 (46.4)	2,560 (43.1)	4,672 (43.4)
**Townsend deprivation (mean)**	-1.4	0.3	0.9
**Educational qualifications**			
CSE/GCSE/A-Level	31,004 (23.0)	1,413 (23.8)	2,003 (18.6)
Degree/Higher/Prof qual	82,369 (61.2)	4,043 (68.0)	7,202 (66.9)
No formal qualifications	21,190 (15.7)	486 (8.2)	1,558 (14.5)
**Ethnic group**			
White British or Irish	133,808 (99.4)	0 (0.0)	0 (0.0)
White Other	755 (0.6)	1,915 (32.2)	2,943 (27.3)
Asian Bangladeshi	0 (0.0)	20 (0.3)	46 (0.4)
Asian Indian	0 (0.0)	824 (13.9)	2,216 (20.6)
Asian Other	0 (0.0)	93 (1.6)	739 (6.9)
Asian Pakistani	0 (0.0)	206 (3.5)	392 (3.6)
Chinese	0 (0.0)	70 (1.2)	471 (4.4)
Black African	0 (0.0)	197 (3.3)	1,284 (11.9)
Black Caribbean	0 (0.0)	1,396 (23.5)	951 (8.8)
Black Other	0 (0.0)	22 (0.4)	30 (0.3)
Mixed	0 (0.0)	816 (13.7)	318 (3.0)
Other	0 (0.0)	383 (6.4)	1,373 (12.8)
**Hearing measures**			
DTT: 'Normal'	119,257 (88.6)	5,331 (89.7)	7,251 (67.4)
DTT: 'Insufficient' or 'Poor'	15,306 (11.4)	611 (10.3)	3,512 (32.6)
Does not use hearing aid	130,507 (97.0)	5,866 (98.7)	10,566 (98.2)
Uses hearing aid	4,059 (3.0)	76 (1.3)	197 (1.8)
No self-reported hearing diff.	96,400 (71.6)	4,779 (80.4)	8,822 (82.0)
Self-reported hearing diff.	38,163 (28.4)	1,163 (19.6)	1,941 (18.0)
No self-reported h/diff. in noise	85,757 (63.7)	4,274 (71.9)	7,362 (68.4)
Self-reported h/diff. in noise	48,806 (36.3)	1,668 (28.1)	3,401 (31.6)

** Mixed group includes "White and Black Caribbean", "White and Black African", "White and Asian" and "Other Mixed"; Other group includes "Arab" and "Any other ethnic groups"*

For peer review only - http://bmjopen.bmj.com/site/about/guidelines.xhtml

RESULTS

Sample characteristics

165,306 participants provided valid hearing test data, reducing to 151,268 after the removal of cases having missing data (Supplementary figure 1). Table 1 shows the characteristics of the sample for each group in the analysis. Levels of 'poor' or 'insufficient' hearing according to the DTT are similar for the White NE and BME NEEM group; however, the later-migrating BME group had over twice the levels of hearing loss according to DTT performance. The two BME groups had similar levels of self-reported hearing difficulty.

Latent variable analysis

A combination of exploratory and confirmatory approaches was used to determine the properties of the hearing health latent classifications. It was expected that there would be a particular group who exhibited poor performance on the DTT, but did not self-report hearing problems or use a hearing aid. To test this, 3-class and 4-class confirmatory models were created with this group specified; other group thresholds were unconstrained, allowing the model to determine their properties. However, this class did not fit well with the data, having very low membership in both models.

Accordingly, an exploratory approach was revisited: unconstrained models with 2 to 7 classes were created, and the model with best fit was selected. A mixture of Bayesian Information Criterion (BIC) and Bootstrapped Likelihood Ratio Test (BLRT) is recommended for determining the optimum number of classes in a latent class analysis [32]; however, the BLRT models for 4 vs 3 classes were not reliable due to a non-positive definite first-order derivative product matrix; as such, the BIC alone was used to determine this number (Supplementary table 2).

Figure 1: Probabilities of 3-class LCA model

Hearing health classes

Figure 1 illustrates the hearing health classes produced in the 3-class model, which were interpreted as follows:

'Normal' (prevalence 60.4%): characterised by low levels of hearing aid use, poor/insufficient hearing on the DTT or reporting hearing difficulty more generally.

'Generally poor' (prevalence 6.9%): those who reported hearing difficulties, the highest levels of poor/insufficient DTT performance and used hearing aids.

'Only subjectively poor' (prevalence 32.7%): those who reported hearing difficulties but had a low probability of having poor/insufficient hearing on the DTT.

For peer review only - http://bmjopen.bmj.com/site/about/guidelines.xhtml

For peer review only

Latent class regression models

A multinomial logistic regression was performed using class membership as the dependent variable. The latent classes were regressed on a range of demographic, socioeconomic and health-related predictors, as well as known biases in the dataset. Uncertainty in the latent class classification was compensated for using the manual "three step" procedure [31]. Predictor variables were introduced hierarchically (see supplementary tables 3-5) to evaluate whether the ethnic inequalities could be explained by the introduction of particular factors.

The latent class regression models described above assumed complete mediation of the relationship between the observed predictor variables and the observed hearing health latent class indicators by the latent classes. This assumption did not tally with our hypotheses, which assumed that responses to the DTT in particular would be different for participants with different values on variables hypothesised to be related to language skills (ethnicity/migration history, language test score and numerical test score) and known biases (whether tested in a mobile centre). We therefore expanded the latent class regression models to include direct, non-mediated effects of these predictors on the DTT outcome in particular. These expanded models were similar in structure to psychometric models of (uniform) "differential item functioning", with the difference that in psychometrics the latent mediator variable is usually a continuously distributed latent factor, whereas in our models it was a discrete latent class distribution. A non-zero estimate for these direct effects would indicate bias, whereby responses to the DTT variable were affected not only by hearing health, but also by other characteristics of the participant, such as language skill and ethnicity. Table 2 shows results for these key variables before and after these direct effects were introduced. A path diagram for the model can be seen in supplementary figure 2.

Without the DTT direct effects, the BME NEEM group had significantly lower odds (OR 0.67, 95% CI 0.53-0.86) of being in the Generally-Poor compared to the normal hearing group; however, these odds were no longer significant (OR 0.83, 95% CI 0.68-1.03) once direct effects were introduced. Higher numeric and language scores caused a significant reduction in the likelihood of having poor or insufficient hearing on the DTT, but did not have a significant impact on group membership. The later-migrating BME group were much more likely than the White NE group to have insufficient or poor hearing (OR 3.85, 95% CI 3.64-4.07).

For peer review only - http://bmjopen.bmj.com/site/about/guidelines.xhtml

Table 2: Coefficients and 95% CI of key variables from multinomial logistic regression

Predictor	Latent hearing health class: Only Subjectively Poor OR (95% CI)	Generally Poor OR (95% CI)	Normal (ref) OR (95% CI)
Excluding DTT direct effects			
BME (NEEM)	0.86 (0.80 to 0.93)***	0.67 (0.53 to 0.86)**	1.00
BME, later-migrating	0.94 (0.89 to 1.00)	0.52 (0.44 to 0.62)***	1.00
Language score: 1 correct	1.01 (0.95 to 1.08)	0.92 (0.81 to 1.05)	1.00
Language score: 2 correct	1.05 (1.00 to 1.11)	0.85 (0.77 to 0.95)**	1.00
Language score: Not taken	0.91 (0.81 to 1.03)	0.86 (0.67 to 1.11)	1.00
Numeric score: >50% correct	1.04 (1.00 to 1.09)	1.02 (0.93 to 1.13)	1.00
Numeric score: Not taken	1.05 (0.94 to 1.17)	1.22 (0.97 to 1.53)	1.00
Mobile test centre	0.98 (0.88 to 1.09)	1.05 (0.83 to 1.32)	1.00
Including DTT direct effects			
BME (NEEM)	0.86 (0.80 to 0.93)***	0.83 (0.68 to 1.03)	1.00
BME, later-migrating	0.94 (0.89 to 1.00)	0.57 (0.49 to 0.67)***	1.00
Language score: 1 correct	1.00 (0.94 to 1.06)	0.98 (0.87 to 1.10)	1.00
Language score: 2 correct	1.04 (0.98 to 1.09)	0.91 (0.83 to 1.00)	1.00
Language score: Not taken	0.89 (0.79 to 1.00)	0.94 (0.76 to 1.17)	1.00
Numeric score: >50% correct	1.04 (0.99 to 1.09)	1.05 (0.96 to 1.15)	1.00
Numeric score: Not taken	1.08 (0.97 to 1.20)	1.10 (0.90 to 1.35)	1.00
Mobile test centre	1.00 (0.90 to 1.12)	0.94 (0.76 to 1.17)	1.00

DTT direct effects	OR of having insufficient or poor hearing
BME (NEEM)	1.04 (0.95 to 1.15)
BME, later-migrating	3.85 (3.64 to 4.07)***
Language score: 1 correct	0.69 (0.65 to 0.74)***
Language score: 2 correct	0.61 (0.58 to 0.65)***
Language score: Not taken	1.12 (1.00 to 1.26)*
Numeric score: >50% correct	0.71 (0.67 to 0.75)***
Numeric score: Not taken	1.34 (1.20 to 1.49)***
Mobile test centre	5.52 (4.99 to 6.10)***

All models controlled for age, sex, education, deprivation score, noise exposure, use of ototoxic medication, medical history, smoking and alcohol use, childhood illnesses and evaluation at a mobile test centre. Asterisk denotes significance: * 95% interval ** 99% interval *** 99.9% interval

For peer review only - http://bmjopen.bmj.com/site/about/guidelines.xhtml

DISCUSSION

This study further investigated the ethnic differences in hearing health observed by Dawes et al. [4] by considering the heterogeneity in language ability of the ethnic minority group, and by validating the results of the DTT against other hearing health outcomes.

By dividing the ethnic minority cohort according to migration age, the present research reveals intergenerational differences in DTT outcomes. As hypothesised, it is the later-migrating BME ethnic minority group who bear the burden of poorer hearing outcomes: the UK-born/early-migrating BME cohort had DTT results that were indistinguishable from the White NE cohort.

Aside from the DTT, the LFA was constructed using self-reported measures, which are thought to be reliable measures of hearing health. This construct indicates that ethnic minority groups are most likely to have normal hearing. However, even after correcting for hypothesised sources of bias, the results from the DTT suggest poorer hearing outcomes for the later-migrating BME group. This is consistent with literature showing ethnic inequalities in other physical and mental health outcomes [3], which have been shown to have multiple causes, including socioeconomic and demographic factors, as well as stress caused by experiences of racial harassment and discrimination [33].

The DTT showed evidence of language bias, having greater dependence upon language ability and migration age than other hearing indicators. The self-report questions did not appear to demonstrate a strong linguistic bias, despite being posed in English. This may mean that the concept of hearing difficulties is reasonably consistently understood, and not prone to misinterpretation due to language barriers.

A secondary finding of this study regards mobile test centres, which caused the largest bias in the DTT, possibly due to elevated background noise levels in the test centres. Respondents who were tested at these centres were 5.52 times (95% CI 4.99-6.10) more likely to have poor/insufficient hearing on the DTT, but were not more likely to have any particular hearing classification. It is also of note that those who did not take the cognitive tests had worse DTT scores. It may be that a cognitive or language difficulty precluded participants from taking, or finishing, these tests.

The LFA produced classes that were substantively consistent with the literature. The likelihood of being in the Generally-Poor hearing group was increased by exposure to noise and most of the health conditions known to affect hearing. The strongest indicator for the Only Subjectively-Poor hearing group was exposure to noise, which seems consistent with a cohort who are concerned about their hearing. Although it may seem unusual that this cohort would perform well on a speech recognition test yet report hearing difficulties in daily life, such phenomena are not uncommon in the literature; explanations include audiometric notches [34], cochlear synaptopathy [35], and health factors unique to the individual [36].

For peer review only - http://bmjopen.bmj.com/site/about/guidelines.xhtml

One limitation to this study regards language measures: proficiency was determined by responses to just two questions, so may be imprecise. Despite this, the measures are still able to provide evidence for the hypothesis of DTT bias, as the LFA outcomes did not exhibit any dependency on language. It is important to note that the DTT is a test of sensorineural hearing loss, detecting only hearing loss of cochlear origin. The DTT, being a test of signal to noise ratio, allows conductive losses to be overcome by increasing the volume of the stimuli, which participants were permitted to do in the UK Biobank test. No other hearing tests were conducted; as such, UK Biobank does not contain any data on conductive pathologies, otoscopy findings, or middle ear function measures.

It must also be noted that the BME classes contain the "White Other" group, which may include participants from Western Europe who are not generally designated as minority ethnic. Regarding generalisability, the UK Biobank, while not employing a representative sample, is thought to be generalisable for disease-exposure relationships [37].

The authors of the Biobank study did not explicitly state why the DTT was used over the Pure-tone Average method, but the available information points to practical factors, such the "minimal impact on throughput" by incorporating the test with the existing touchscreen questionnaire [38], and no requirement for calibrated equipment or a sound-treated test booth. Unfortunately, use of the DTT may have resulted in a test that is biased for non-native English speakers.

Even after accounting for this bias, the results suggest that there are still ethnic inequalities in hearing health, albeit with contradiction between psychophysical and self-reported outcomes. Designers of future studies must take precautions to ensure that this inequality can be reliably analysed by ensuring that hearing test measures are not language-dependent. The present study may also have implications for hearing screening applications, which many countries currently implement using the DTT [39]. Digit Triplet Tests are being translated to an increasing number of languages [40], although it may be some time before tests are available in all 6,500 languages spoken globally. Until such time, non-linguistic hearing tests may be the most practical solution to lessen the dependency on language proficiency in psychophysical measures of hearing.

For peer review only

FUNDING

This work was supported by the Economic and Social Research Council [grant number ES/P000665/1] and the NIHR Manchester Biomedical Research Centre.

ACKNOWLEDGEMENTS

We would like to express our gratitude to Dr. Antje Heinrich for her useful suggestions in response to a presentation on the initial stages of this work. We would also like to thank the BMJ Open editors and external reviewers for their thoughtful comments and suggestions.

For peer review only - http://bmjopen.bmj.com/site/about/guidelines.xhtml

CONTRIBUTORS

Mr. Taylor carried out the conception and design of the study, analysis and interpretation of results, and drafting of the manuscript. Dr. Shryane, Dr. Kapadia, Dr. Dawes and Dr. Norman made substantial contributions to the conception and design of the study, and interpretation of data, as well as critically revising the manuscript. All authors approved the final version and agree to be accountable for all aspects of the work.

COMPETING INTERESTS

None declared.

DATA AVAILABILITY STATEMENT

UK Biobank data is held in an open access resource available to researchers via the procedure described at http://www.ukbiobank.ac.uk/using-the-resource/.

FIGURE LEGENDS

Table 1: Sample characteristics (N=151,268). Values are number (percentage).

Table 2: Coefficients and 95% CI of key variables from multinomial logistic regression

Figure 1: Probabilities of 3-class LCA model

Supplementary Table 1: *SRT variation by test centre*

Supplementary Table 2: BIC for exploratory latent class analysis (step 1)

Supplementary Table 3: *Normal hearing* group Vs *Only Subjectively-Poor* hearing group

Supplementary Table 4: *Normal hearing* group Vs *Generally-Poor hearing group*

Supplementary Table 5: *DTT pathway*

Supplementary Figure 1: Derivation of final sample size

Supplementary Figure 2: Model path diagram. In the diagram, boxes A-E are predictors of hearing health. α represents the latent classes of hearing health. Boxes 1-4 are indicator variables for latent hearing health. Dotted lines represent the direct effect paths.

For peer review only

REFERENCES

1 Vos T, Allen C, Arora M, *et al.* Global, regional, and national incidence, prevalence, and years lived with disability for 310 diseases and injuries, 1990–2015: a systematic analysis for the Global Burden of Disease Study 2015. *Lancet* 2016;**388**:1545–602. doi:10.1016/S0140-6736(16)31678-6

2 Action on Hearing Loss. Facts and figures on hearing loss, deafness and tinnitus, A1205/0416. 2016.

3 Department of Health. Tackling health inequalities: 10 years on a review of developments in tackling health inequalities in England over the last 10 years. 2009.

4 Dawes P, Fortnum H, Moore DR, *et al.* Hearing in middle age: a population snapshot of 40–69 year olds in the UK. *Ear Hear* 2014;**35**:e44–51. doi:10.1097/AUD.0000000000000010

5 Bécares L. Which ethnic groups have the poorest health? Ethnic health inequalities 1991 to 2011. 2013. http://www.ethnicity.ac.uk/medialibrary/briefingsupdated/which-ethnic-groups-have-the-poorest-health.pdf

6 Agrawal Y, Platz EA, Niparko JK. Prevalence of Hearing Loss and Differences by Demographic Characteristics Among US Adults: Data From the National Health and Nutrition Examination Survey, 1999-2004. *Arch Intern Med* 2008;**168**:1522. doi:10.1001/archinte.168.14.1522

7 NatCen. Nurse project instructions P8814. 2014. http://doc.ukdataservice.ac.uk/doc/7919/mrdoc/pdf/7919_hse2014_supporting_documents.pdf

8 NatCen. ELSA Wave 7 Mainstage Interviewer Project Instructions. 2014.

9 Jansen S, Luts H, Wagener KC, *et al.* The French digit triplet test: a hearing screening tool for speech intelligibility in noise. *Int J Audiol* 2010;**49**:378–87. doi:10.3109/14992020903431272

10 Mayo LH, Florentine M, Buus S. Age of second-language acquisition and perception of speech in noise. *J Speech Lang Hear Res* 1997;**40**:686–93.http://www.ncbi.nlm.nih.gov/pubmed/9210123

11 Warzybok A, Wagener KC, Brand T, *et al.* Influence of the linguistic complexity in relation to speech material on non-native speech perception in noise. In: *DAGA 2010 - Berlin*. 2010.

12 Allen N, Sudlow C, Downey P, *et al.* UK Biobank: Current status and what it means for epidemiology. *Heal Policy Technol* 2012;**1**:123–6. doi:10.1016/j.hlpt.2012.07.003

13 von Elm E, Altman DG, Egger M, *et al.* The strengthening the reporting of observational studies in epidemiology (STROBE) statement: Guidelines for reporting observational studies. *Int J Surg* Published Online First: 2014. doi:10.1016/j.ijsu.2014.07.013

14 Smits C, Kapteyn TS, Houtgast T. Development and validation of an automatic speech-in-noise screening test by telephone. *Int J Audiol* 2004;**43**:15–28.http://www.ncbi.nlm.nih.gov/pubmed/14974624

15 Kramer SE, Kapteyn TS, Festen JM, *et al.* The self-reported handicapping effect of hearing disabilities. *Int J Audiol* Published Online First: 1998. doi:10.3109/00206099809072984

16 Hall SJ. *The Development of a New English Sentence In Noise Test and an English Number Recognition Test.* 2006.

17 Corso JF. Age and sex differences in pure-tone thresholds. Survey of hearing levels from 18 to 65 years. *Arch Otolaryngol* Published Online First: 1963. doi:10.1001/archotol.1963.00750010399008

18 Baraldi GDS, De Almeida LC, Borges ACDC. Hearing loss in aging. *Braz J Otorhinolaryngol* 2007;**73**:64–70.

For peer review only - http://bmjopen.bmj.com/site/about/guidelines.xhtml

For peer review only

doi:10.1016/s1808-8694(15)31123-x

19 Rigters SC, Metselaar M, Wieringa MH, *et al.* Contributing Determinants to Hearing Loss in Elderly Men and Women: Results from the Population-Based Rotterdam Study. *Audiol Neurotol* 2016;**21**:10–5. doi:10.1159/000448348

20 Khil L, Wellmann J, Berger K. Determinants of Single and Multiple Sensory Impairments in an Urban Population. *Otolaryngol Neck Surg* 2015;**153**:364–71. doi:10.1177/0194599815588913

21 Popelka MM, Cruickshanks KJ, Wiley TL, *et al.* Moderate alcohol consumption and hearing loss: a protective effect. *J Am Geriatr Soc* 2000;**48**:1273–8.http://www.ncbi.nlm.nih.gov/pubmed/11037015

22 Department of Health. UK Chief Medical Officers' Low Risk Drinking Guidelines. 2016. https://www.gov.uk/government/uploads/system/uploads/attachment_data/file/545937/UK_CMOs__report.pdf

23 Kuo C-L, Shiao A-S, Wang S-J, *et al.* Risk of sudden sensorineural hearing loss in stroke patients: A 5-year nationwide investigation of 44,460 patients. *Medicine (Baltimore)* 2016;**95**:e4841. doi:10.1097/MD.0000000000004841

24 Schacht J, Talaska AE, Rybak LP. Cisplatin and aminoglycoside antibiotics: hearing loss and its prevention. *Anat Rec (Hoboken)* 2012;**295**:1837–50. doi:10.1002/ar.22578

25 Duthey B. *Background Paper 6.21: Hearing Loss.* 2013.https://www.who.int/medicines/areas/priority_medicines/BP6_21Hearing.pdf

26 Fortnum HM. Hearing impairment after bacterial meningitis: A review. Arch. Dis. Child. 1992. doi:10.1136/adc.67.9.1128

27 Cohen BE, Durstenfeld A, Roehm PC. Viral causes of hearing loss: A review for hearing health professionals. *Trends Hear* Published Online First: 2014. doi:10.1177/2331216514541361

28 Yuan J, Sun Y, Sang S, *et al.* The risk of cognitive impairment associated with hearing function in older adults: A pooled analysis of data from eleven studies. *Sci Rep* 2018;**8**:1–10. doi:10.1038/s41598-018-20496-w

29 Nazroo JY. *Ethnicity And Mental Health: Findings From A National Community Survey.* Policy Studies Institute 1999.

30 UK Biobank EGC. Minutes of UK Biobank Ethics and Governance Council Twenty Third Meeting. 2010. https://egcukbiobank.org.uk/sites/default/files/meetings/EGC23 agenda and report.pdf

31 Asparouhov T, Muthén B. Auxiliary Variables in Mixture Modeling: Three-Step Approaches Using Mplus. *Struct Equ Model* Published Online First: 2014. doi:10.1080/10705511.2014.915181

32 Nylund KL, Asparouhov T, Muthén BO. Deciding on the number of classes in latent class analysis and growth mixture modeling: A Monte Carlo simulation study. *Struct Equ Model* Published Online First: 2007. doi:10.1080/10705510701575396

33 Nazroo JY. The structuring of ethnic inequalities in health: economic position, racial discrimination, and racism. *Am J Public Health* 2003;**93**:277–84.http://www.ncbi.nlm.nih.gov/pubmed/12554585 (accessed 27 Jun 2017).

34 Zhao F, Stephens D. Audioscan testing in patients with King-Kopetzky syndrome. *Acta Otolaryngol* Published Online First: 1999. doi:10.1080/00016489950181297

35 Bharadwaj HM, Masud S, Mehraei G, *et al.* Individual differences reveal correlates of hidden hearing deficits. *J Neurosci* Published Online First: 2015. doi:10.1523/JNEUROSCI.3915-14.2015

For peer review only

36 Pronk M, Deeg DJH, Kramer SE. Explaining discrepancies between the digit triplet speech-in-noise test score and self-reported hearing problems in older adults. *J Speech, Lang Hear Res* Published Online First: 2018. doi:10.1044/2018_JSLHR-H-17-0124

37 Fry A, Littlejohns TJ, Sudlow C, *et al.* Comparison of Sociodemographic and Health-Related Characteristics of UK Biobank Participants with the General Population. *Am J Epidemiol* Published Online First: 21 June 2017. doi:10.1093/aje/kwx246

38 UK Biobank. UK Biobank Ethics and Governance Council Report - Fifteenth Meeting. 2008.

39 Watson CS, Kidd GR, Miller JD, *et al.* Telephone screening tests for functionally impaired hearing: Current use in seven countries and development of a US version. *J Am Acad Audiol* Published Online First: 2012. doi:10.3766/jaaa.23.10.2

40 Zokoll MA, Hochmuth S, Warzybok A, *et al.* Speech-in-noise tests for multilingual hearing screening and diagnostics. *Am J Audiol* Published Online First: 2013. doi:10.1044/1059-0889(2013/12-0061)

For peer review only

SUPPLEMENTARY FIGURES

Supplementary Table 1: *SRT variation by test centre*

Supplementary Table 2: BIC for exploratory latent class analysis (step 1)

Supplementary Table 3: *Normal hearing* group Vs *Only Subjectively-Poor* hearing group

Supplementary Table 4: *Normal hearing* group Vs *Generally-Poor hearing group*

Supplementary Table 5: *DTT pathway*

Supplementary Figure 1: Derivation of final sample size

Supplementary Figure 2: Model path diagram

For peer review only - http://bmjopen.bmj.com/site/about/guidelines.xhtml

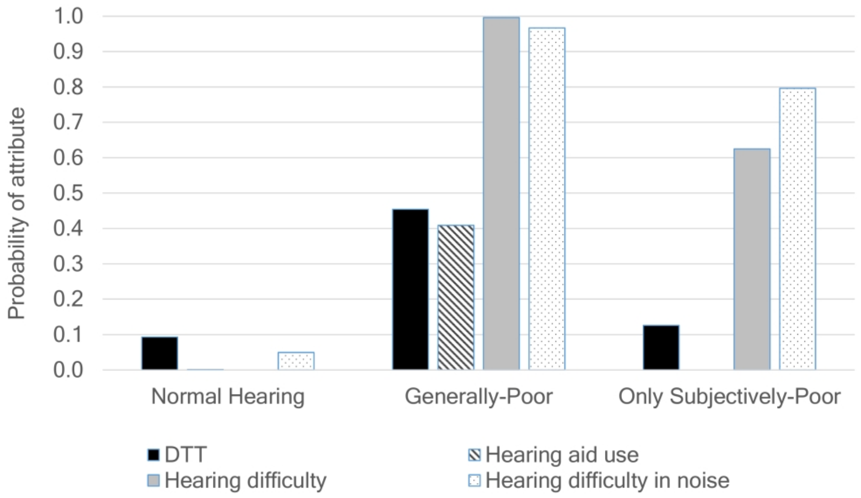

Figure 1: Probabilities of 3-class LCA model

297x209mm (300 x 300 DPI)

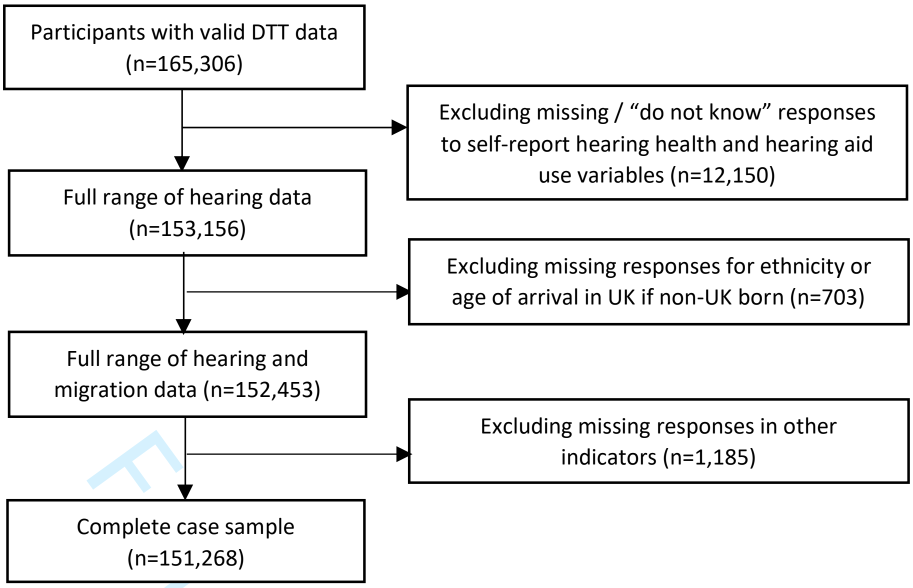
Participants with valid DTT data (n=165,306)
Excluding missing / “do not know” responses to self-report hearing health and hearing aid use variables (n=12,150)
Full range of hearing data (n=153,156)
Excluding missing responses for ethnicity or age of arrival in UK if non-UK born (n=703)
Full range of hearing and migration data (n=152,453)
Excluding missing responses in other indicators (n=1,185)
Complete case sample (n=151,268)

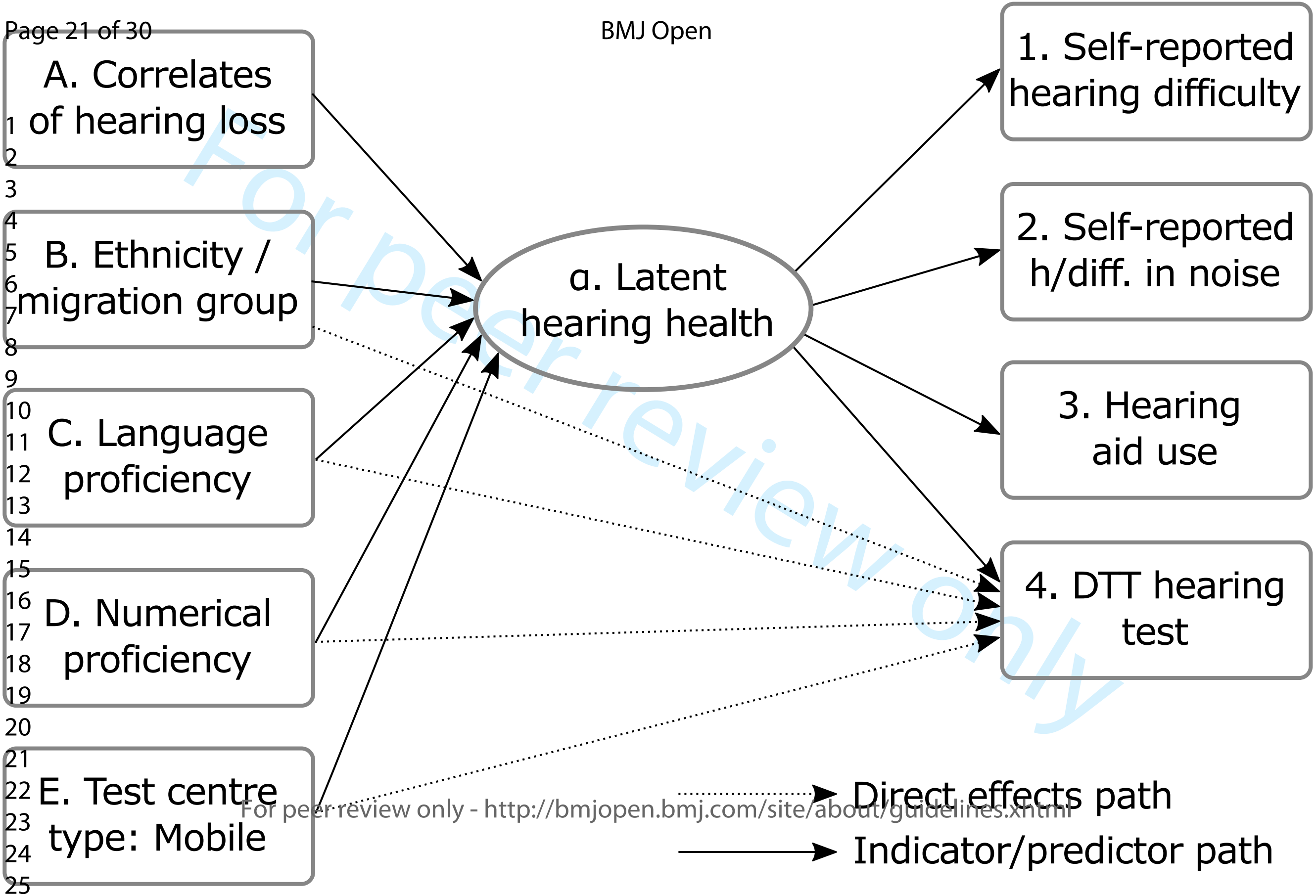
A. Correlates of hearing loss
B. Ethnicity / migration group
C. Language proficiency
D. Numerical proficiency
E. Test centre type: Mobile
ɑ. Latent hearing health
1. Self-reported hearing difficulty
2. Self-reported h/diff. in noise
3. Hearing aid use
4. DTT hearing test
Direct effects path
Indicator/predictor path

Test centre	Average SRT Mean	Standard Deviation
Birmingham	-7.1	1.8
Bristol	-7.1	1.6
Croydon	-7.0	1.8
Hounslow	-7.4	1.9
Liverpool	-7.5	1.7
Middlesbrough	-7.5	1.9
Nottingham	-6.8	1.9
Sheffield	-7.7	1.6
Swansea	-6.0	1.8
Wrexham	-6.0	1.9

For peer review only

No. classes	BIC
2	456,790
3	455,467
4	455,498
5	455,557
6	455,617
7	455,676

For peer review only

	m1	m2	m3	m4	m5
**Covariate**	**Est**	**Est**	**Est**	**Est**	**Est**
Age	1.03 (1.03 to 1.03)	1.03 (1.03 to 1.03)	1.04 (1.04 to 1.04)	1.04 (1.04 to 1.04)	1.03 (1.03 to 1.04)
Sex: Male	1.71 (1.66 to 1.75)	1.69 (1.64 to 1.74)	1.36 (1.32 to 1.41)	1.36 (1.32 to 1.41)	1.37 (1.33 to 1.41)
BME (NEEM)	0.88 (0.82 to 0.95)	0.86 (0.80 to 0.92)	0.86 (0.80 to 0.92)	0.86 (0.80 to 0.93)	0.84 (0.78 to 0.91)
BME, later-migrating	0.90 (0.85 to 0.95)	0.86 (0.81 to 0.91)	0.91 (0.86 to 0.97)	0.94 (0.89 to 1.00)	0.94 (0.88 to 1.00)
Mobile test centre	1.00 (0.90 to 1.12)	1.00 (0.90 to 1.11)	0.98 (0.88 to 1.09)	0.98 (0.88 to 1.09)	1.00 (0.90 to 1.12)
Education: High School		0.88 (0.84 to 0.93)	0.99 (0.94 to 1.04)	0.97 (0.92 to 1.02)	1.00 (0.95 to 1.05)
Education: Degree		0.99 (0.95 to 1.03)	1.13 (1.08 to 1.17)	1.10 (1.05 to 1.15)	1.14 (1.09 to 1.20)
Townsend dep. Score		1.02 (1.01 to 1.02)	1.00 (1.00 to 1.01)	1.01 (1.00 to 1.01)	1.00 (1.00 to 1.01)
Exposure to loud noise			1.84 (1.78 to 1.91)	1.85 (1.78 to 1.91)	1.85 (1.78 to 1.91)
Exposure to loud music			1.82 (1.74 to 1.89)	1.82 (1.75 to 1.89)	1.81 (1.74 to 1.89)
Use of ototoxic meds.			1.05 (1.01 to 1.10)	1.05 (1.01 to 1.10)	1.06 (1.01 to 1.11)
Stroke			1.07 (0.94 to 1.21)	1.07 (0.94 to 1.22)	1.00 (0.87 to 1.14)
Diabetes			1.03 (0.96 to 1.10)	1.03 (0.97 to 1.10)	1.02 (0.95 to 1.09)
Cardiovascular illness			1.12 (1.06 to 1.19)	1.12 (1.06 to 1.19)	1.13 (1.07 to 1.19)
Hypertension			0.93 (0.90 to 0.96)	0.93 (0.90 to 0.96)	0.93 (0.90 to 0.95)
Smoker (at some stage)			1.11 (1.07 to 1.14)	1.11 (1.07 to 1.14)	1.11 (1.07 to 1.14)
Alcohol: Former			1.10 (1.01 to 1.20)	1.10 (1.01 to 1.20)	1.08 (0.99 to 1.18)
Alcohol: Meets guidelines			1.00 (0.95 to 1.05)	0.99 (0.94 to 1.04)	1.01 (0.96 to 1.06)
Alcohol: Exceeds g/l			1.02 (0.96 to 1.07)	1.01 (0.96 to 1.06)	1.02 (0.97 to 1.08)
Meningitis as child			1.62 (1.12 to 2.35)	1.62 (1.11 to 2.35)	1.62 (1.13 to 2.32)
MMR as child			1.02 (0.90 to 1.16)	1.02 (0.89 to 1.16)	0.98 (0.86 to 1.12)
Language score: 1 correct				1.01 (0.95 to 1.08)	1.00 (0.94 to 1.06)
Language score: 2 correct				1.05 (1.00 to 1.11)	1.04 (0.98 to 1.09)
Language score: Not taken				0.91 (0.81 to 1.03)	0.89 (0.79 to 1.00)
Numeric score: >50% correct				1.04 (1.00 to 1.09)	1.04 (0.99 to 1.09)
Numeric score: Not taken				1.05 (0.94 to 1.17)	1.08 (0.97 to 1.20)

For peer review only

Covariate	m1 Est	m2 Est	m3 Est	m4 Est	m5 Est
Age	1.13 (1.12 to 1.14)	1.13 (1.12 to 1.13)	1.13 (1.13 to 1.14)	1.13 (1.13 to 1.14)	1.15 (1.15 to 1.16)
Sex: Male	1.93 (1.82 to 2.06)	1.93 (1.81 to 2.06)	1.36 (1.27 to 1.46)	1.36 (1.26 to 1.46)	1.33 (1.25 to 1.42)
BME (NEEM)	0.74 (0.58 to 0.94)	0.72 (0.56 to 0.91)	0.68 (0.54 to 0.87)	0.67 (0.53 to 0.86)	0.83 (0.68 to 1.03)
BME, later-migrating	0.58 (0.50 to 0.68)	0.55 (0.47 to 0.65)	0.55 (0.47 to 0.65)	0.52 (0.44 to 0.62)	0.57 (0.49 to 0.67)
Mobile test centre	1.13 (0.90 to 1.41)	1.12 (0.90 to 1.40)	1.05 (0.84 to 1.32)	1.05 (0.83 to 1.32)	0.94 (0.76 to 1.17)
Education: High School		0.63 (0.57 to 0.69)	0.80 (0.72 to 0.88)	0.83 (0.75 to 0.91)	0.80 (0.73 to 0.87)
Education: Degree		0.71 (0.66 to 0.77)	0.90 (0.83 to 0.98)	0.94 (0.87 to 1.03)	0.86 (0.80 to 0.93)
Townsend dep. Score		1.03 (1.02 to 1.04)	1.01 (0.99 to 1.02)	1.01 (0.99 to 1.02)	1.02 (1.01 to 1.03)
Exposure to loud noise			2.89 (2.69 to 3.10)	2.86 (2.67 to 3.07)	2.77 (2.61 to 2.95)
Exposure to loud music			2.15 (1.95 to 2.37)	2.14 (1.94 to 2.36)	2.13 (1.95 to 2.32)
Use of ototoxic meds.			1.16 (1.06 to 1.27)	1.16 (1.06 to 1.27)	1.13 (1.05 to 1.22)
Stroke			1.15 (0.93 to 1.42)	1.14 (0.92 to 1.42)	1.33 (1.11 to 1.60)
Diabetes			1.21 (1.07 to 1.36)	1.20 (1.07 to 1.36)	1.24 (1.12 to 1.37)
Cardiovascular illness			1.24 (1.12 to 1.37)	1.24 (1.12 to 1.37)	1.20 (1.10 to 1.31)
Hypertension			0.93 (0.87 to 1.00)	0.93 (0.87 to 1.00)	0.95 (0.89 to 1.01)
Smoker (at some stage)			1.13 (1.06 to 1.21)	1.14 (1.06 to 1.21)	1.13 (1.07 to 1.20)
Alcohol: Former			1.04 (0.87 to 1.25)	1.05 (0.87 to 1.26)	1.14 (0.97 to 1.33)
Alcohol: Meets guidelines			1.01 (0.90 to 1.12)	1.01 (0.91 to 1.13)	0.95 (0.87 to 1.05)
Alcohol: Exceeds g/l			0.81 (0.72 to 0.91)	0.82 (0.73 to 0.92)	0.79 (0.72 to 0.88)
Meningitis as child			2.93 (1.59 to 5.41)	2.95 (1.60 to 5.43)	2.88 (1.64 to 5.08)
MMR as child			1.12 (0.84 to 1.49)	1.13 (0.84 to 1.51)	1.29 (1.01 to 1.65)
Language score: 1 correct				0.92 (0.81 to 1.05)	0.98 (0.87 to 1.10)
Language score: 2 correct				0.85 (0.77 to 0.95)	0.91 (0.83 to 1.00)
Language score: Not taken				0.86 (0.67 to 1.11)	0.94 (0.76 to 1.17)
Numeric score: >50% correct				1.02 (0.93 to 1.13)	1.05 (0.96 to 1.15)
Numeric score: Not taken				1.22 (0.97 to 1.53)	1.10 (0.90 to 1.35)

For peer review only

DTT pathway	m1<br>Est	m2<br>Est	m3<br>Est	m4<br>Est	m5<br>Est
BME, migrated age <12					1.04 (0.95 to 1.15)
BME, migrated age >= 12					3.85 (3.64 to 4.07)
Language score: 1 correct					0.69 (0.65 to 0.74)
Language score: 2 correct					0.61 (0.58 to 0.65)
Language score: Not taken					1.12 (1.00 to 1.26)
Numeric score: >50% correct					0.71 (0.67 to 0.75)
Numeric score: Not taken					1.34 (1.20 to 1.49)
Mobile test centre					5.52 (4.99 to 6.10)

For peer review only

Reporting checklist for cross sectional study.

Based on the STROBE cross sectional guidelines.

Instructions to authors

Complete this checklist by entering the page numbers from your manuscript where readers will find each of the items listed below.

Your article may not currently address all the items on the checklist. Please modify your text to include the missing information. If you are certain that an item does not apply, please write "n/a" and provide a short explanation.

Upload your completed checklist as an extra file when you submit to a journal.

In your methods section, say that you used the STROBE cross sectionalreporting guidelines, and cite them as:

von Elm E, Altman DG, Egger M, Pocock SJ, Gotzsche PC, Vandenbroucke JP. The Strengthening the Reporting of Observational Studies in Epidemiology (STROBE) Statement: guidelines for reporting observational studies.

For peer review only

		Reporting Item	Page Number
**Title and abstract**			
Title	#1a	Indicate the study's design with a commonly used term in the title or the abstract	2
Abstract	#1b	Provide in the abstract an informative and balanced summary	2

		of what was done and what was found	
**Introduction**			
Background / rationale	#2	Explain the scientific background and rationale for the investigation being reported	3
Objectives	#3	State specific objectives, including any prespecified hypotheses	3-4
**Methods**			
Study design	#4	Present key elements of study design early in the paper	4
Setting	#5	Describe the setting, locations, and relevant dates, including periods of recruitment, exposure, follow-up, and data collection	4
Eligibility criteria	#6a	Give the eligibility criteria, and the sources and methods of selection of participants.	4
	#7	Clearly define all outcomes, exposures, predictors, potential confounders, and effect modifiers. Give diagnostic criteria, if applicable	4-6
Data sources / measurement	#8	For each variable of interest give sources of data and details of methods of assessment (measurement). Describe comparability of assessment methods if there is more than one group. Give information separately for for exposed and unexposed groups if applicable.	4-6
Bias	#9	Describe any efforts to address potential sources of bias	5-6

For peer review only

Study size	#10	Explain how the study size was arrived at	8
Quantitative variables	#11	Explain how quantitative variables were handled in the analyses. If applicable, describe which groupings were chosen, and why	4-6
Statistical methods	#12a	Describe all statistical methods, including those used to control for confounding	6
Statistical methods	#12b	Describe any methods used to examine subgroups and interactions	N/A
Statistical methods	#12c	Explain how missing data were addressed	8
Statistical methods	#12d	If applicable, describe analytical methods taking account of sampling strategy	N/A
Statistical methods	#12e	Describe any sensitivity analyses	N/A
**Results**			
Participants	#13a	Report numbers of individuals at each stage of study—eg numbers potentially eligible, examined for eligibility, confirmed eligible, included in the study, completing follow-up, and analysed. Give information separately for for exposed and unexposed groups if applicable.	8 (supp fig. 1)
Participants	#13b	Give reasons for non-participation at each stage	8 (supp fig. 1)

For peer review only

Participants	#13c	Consider use of a flow diagram	8 (supp fig. 1)
Descriptive data	#14a	Give characteristics of study participants (eg demographic, clinical, social) and information on exposures and potential confounders. Give information separately for exposed and unexposed groups if applicable.	7
Descriptive data	#14b	Indicate number of participants with missing data for each variable of interest	N/A
Outcome data	#15	Report numbers of outcome events or summary measures. Give information separately for exposed and unexposed groups if applicable.	N/A
Main results	#16a	Give unadjusted estimates and, if applicable, confounder-adjusted estimates and their precision (eg, 95% confidence interval). Make clear which confounders were adjusted for and why they were included	8-10 (supp. tbl. 4-6)
Main results	#16b	Report category boundaries when continuous variables were categorized	N/A
Main results	#16c	If relevant, consider translating estimates of relative risk into absolute risk for a meaningful time period	N/A
Other analyses	#17	Report other analyses done—e.g., analyses of subgroups and interactions, and sensitivity analyses	N/A

Discussion

For peer review only

Key results	#18	Summarise key results with reference to study objectives	11-12
Limitations	#19	Discuss limitations of the study, taking into account sources of potential bias or imprecision. Discuss both direction and magnitude of any potential bias.	11-12
Interpretation	#20	Give a cautious overall interpretation considering objectives, limitations, multiplicity of analyses, results from similar studies, and other relevant evidence.	11-12
Generalisability	#21	Discuss the generalisability (external validity) of the study results	12
**Other Information**			
Funding	#22	Give the source of funding and the role of the funders for the present study and, if applicable, for the original study on which the present article is based	12

Notes:

- 13a: 8 (supp fig. 1)
- 13b: 8 (supp fig. 1)
- 13c: 8 (supp fig. 1) The STROBE checklist is distributed under the terms of the Creative Commons Attribution License CC-BY. This checklist was completed on 22. February 2020 using https://www.goodreports.org/, a tool made by the EQUATOR Network in collaboration with Penelope.ai

For peer review only
